# Inhibition of DNA Methylation in *Picochlorum soloecismus* Alters Algae Productivity

**DOI:** 10.3389/fgene.2020.560444

**Published:** 2020-10-15

**Authors:** Christina R. Steadman, Shounak Banerjee, Yuliya A. Kunde, Claire K. Sanders, Babetta L. Marrone, Scott N. Twary

**Affiliations:** Los Alamos National Laboratory, Bioenergy and Biome Sciences, Los Alamos, NM, United States

**Keywords:** algae, epigenetics, 5-aza-2′-deoxycytidine, DNA methylation, lipid accumulation, fatty acid synthesis, bisulfite sequencing

## Abstract

Eukaryotic organisms regulate the organization, structure, and accessibility of their genomes through chromatin remodeling that can be inherited as epigenetic modifications. These DNA and histone protein modifications are ultimately responsible for an organism’s molecular adaptation to the environment, resulting in distinctive phenotypes. Epigenetic manipulation of algae holds yet untapped potential for the optimization of biofuel production and bioproduct formation; however, epigenetic machinery and modes-of-action have not been well characterized in algae. We sought to determine the extent to which the biofuel platform species *Picochlorum soloecismus* utilizes DNA methylation to regulate its genome. We found candidate genes with domains for DNA methylation in the *P. soloecismus* genome. Whole-genome bisulfite sequencing revealed DNA methylation in all three cytosine contexts (CpG, CHH, and CHG). While global DNA methylation is low overall (∼1.15%), it occurs in appreciable quantities (12.1%) in CpG dinucleotides in a bimodal distribution in all genomic contexts, though terminators contain the greatest number of CpG sites per kilobase. The *P. soloecismus* genome becomes hypomethylated during the growth cycle in response to nitrogen starvation. Algae cultures were treated daily across the growth cycle with 20 μM 5-aza-2′-deoxycytidine (5AZA) to inhibit propagation of DNA methylation in daughter cells. 5AZA treatment significantly increased optical density and forward and side scatter of cells across the growth cycle (16 days). This increase in cell size and complexity correlated with a significant increase (∼66%) in lipid accumulation. Site specific CpG DNA methylation was significantly altered with 5AZA treatment over the time course, though nitrogen starvation itself induced significant hypomethylation in CpG contexts. Genes involved in several biological processes, including fatty acid synthesis, had altered methylation ratios in response to 5AZA; we hypothesize that these changes are potentially responsible for the phenotype of early induction of carbon storage as lipids. This is the first report to utilize epigenetic manipulation strategies to alter algal physiology and phenotype. Collectively, these data suggest these strategies can be utilized to fine-tune metabolic responses, alter growth, and enhance environmental adaption of microalgae for desired outcomes.

## Introduction

Eukaryotic organisms control the organization and accessibly of their genomes via covalent modification of DNA and chromatin proteins. These modifications are collectively referred to as epigenetic modifications, which, under the purview of strict scrutiny, are reversible and yet heritable during mitotic activity (Feng et al., 2010b). Epigenetic mechanisms regulate a plethora of processes in mammalian and plant species, ranging from the fidelity of DNA replication, repair, and protection to DNA transcription and expression (Jaenisch and Bird, 2003). These processes are globally defined as either (1) covalent modification of basic amino acids located in the N-terminal domain of histone proteins that comprise nucleosome structures (i.e., histone modifications) or (2) covalent modification of the nucleic acids, adenosine or cytosine (i.e., DNA modifications). In plants, methylation of cytosines in DNA can occur in multiple genomic regions and dinucleotide contexts, including CpG, CHH, and CHG (where H corresponds to A, T or C). This DNA methylation is important for plant growth and dynamic responses to environmental perturbations and directly influences the plant’s phenotype (Zhang et al., 2018).

Microalgae are photosynthetic, single-celled eukaryotes. Many microalgae species have relatively small genomes, particularly in comparison with humans and polyploid plant species. Of the thousands of algae species, very few have been sequenced, and even fewer have had their epigenomes measured (Blaby-Haas and Merchant, 2019). The model algae organism, *Chlamydomonas reinhardtii*, has been used extensively to study the mechanisms of epigenetic regulation, inheritance, and adaption (Cerutti, 1997; van Dijk et al., 2005; Shaver et al., 2010; Pandey et al., 2012; Fu et al., 2015; Kronholm et al., 2017). However, unlike mammalian species, in which the presence and functionality of epigenetic modifications is similar among several species, patterns of epigenetic modifications (and even function) have proven to be dissimilar (or not even present) in algae (Tirichine and Bowler, 2011; Veluchamy et al., 2014; Tirichine et al., 2017). This is likely attributed to either evolutionary divergence of algae and/or variable genome size. Organisms with smaller genomes use other mechanisms of genomic control, including operons and RNA interference (RNAi), both of which alter gene expression without the need for chromatin remodeling processes. Interestingly, despite the lack of differentiation and the relative compactness of their genomes, many microalgae tend to utilize some form of epigenetic modification, though relatively few have been tested (Müller et al., 1990; Umen and Goodenough, 2001; Babinger et al., 2007; Zemach et al., 2010; Lopez et al., 2015; Ngan et al., 2015). Thus, given the breadth of genetic diversity among microalgae, these organisms provide an opportunity to study the evolution of epigenetic mechanisms. However, this diversity requires that each modification must be assessed under environmental variability for each species of interest to determine the presence and function of epigenetic modifications in microalgae collectively.

We sought to determine the relative importance of DNA modifications, particularly 5-methylcytosine, for our microalgae species of interest, *Picochlorum soloecismus*, which has a small haploid genome (15.6 Mb) (Gonzalez-Esquer et al., 2018). We are interested in the phenotype of this species, particularly under nutrient-limited conditions that induce carbon sequestration into lipid and carbohydrate molecules. This “carbon accumulation” phenotype under duress has potential applications for the production of biofuels and other bioproducts (Alishah Aratboni et al., 2019). A recent algae biofuel consortium (the National Alliance for Advanced Biofuels and Bioproducts) denoted *P. soloecismus* as a promising feedstock for biofuel research (Unkefer et al., 2017). The *Picochlorum* genus is highly adaptive to environmental variation in salinity, temperature, pH, and nutrients; it readily alters its gene expression as such to induce particular phenotypes under these various conditions (Foflonker et al., 2015; Krasovec et al., 2018; Dahlin et al., 2019; Gonzalez-Esquer et al., 2019; Steadman Tyler et al., 2019). Bioengineering *P. soloecismus* includes the manipulation of gene expression to mimic environmental conditions that drive carbon sequestration, but efforts have been limited. Understanding the mechanisms by which this organism controls its genome is thus useful for maximizing its productivity. To aid in this challenge, we sought to quantify DNA methylation and determine its influence on the physiology and phenotype of *P. soloecismus*.

We used treatment with 5-aza-2′-deoxycytidine (5AZA) in cultivation of *P. soloecismus* to inhibit the formation of 5-methylcytosine (5mC) DNA methylation under baseline environmental conditions and during nitrogen starvation. This treatment inhibits binding of DNA methyltransferase enzymes to hemimethylated DNA during replication, thereby interfering with maintenance methylation on the lagging strand (Christman, 2002). After mitosis, daughter cells lack this epigenetic modification, and over the course of growth, each new cell has less 5mC DNA methylation (typically halved in each subsequent generation of cells). In mammalian cells, this treatment induces cell cycle arrest and apoptosis, thus demonstrating the importance of DNA methylation for maintaining cell function and physiology (Palii et al., 2008). Here, we report that 5mC DNA methylation occurs primarily in CpG contexts in *P. soloecismus*, though it was also found in CHG and CHH contexts. The relative abundance of DNA methylation is low but occurs in multiple genomic loci, including gene bodies, promoters, terminators, and intergenic regions. DNA methylation in *P. soloecismus* is dynamic and responsive over the algal growth cycle. Inhibition of 5mC propagation resulted in altered cell growth and increased lipid accumulation, suggesting this epigenetic modification has physiological relevance and control of the *P. soloecismus* stress phenotype. This study suggests that epigenetic manipulation of algal DNA methylomes may allow for fine-tuning metabolic responses, alteration of growth, and enhanced environmental adaption for biofuel and bioproduct outcomes.

## Materials and Methods

### Data Mining for DNA Methyltransferase Genes in the *P. soloecismus* Genome

Using methods previously described, we interrogated the *P. soloecismus* genome for genes encoding epigenetic machinery with the capacity for DNA methylation (Hovde et al., 2018). Briefly, queries of known DNA methylation protein sequences were tested against the *P. soloecismus* protein sequence data. Sequences with similar homology were queried using BLASTP (Altschul et al., 1990) and for specific Pfam domains (El-Gebali et al., 2019). The presence of domains was confirmed in the annotated *P. soloecismus* genome using Pfam and InterPro domains considered essential for epigenetic function in each protein (Mitchell et al., 2019).

### Microalgae Cultivation

For DNA methylation experiments, *P. soloecismus* was cultivated as previously described (Steadman Tyler et al., 2019). Briefly, cells were grown in 250 mL shaker flasks, maintained at ambient temperature, under 300 μmolm^–2^s^–1^ fluorescent light with a 16 h/8 h light:dark cycle in modified f/2 media with 8.8 mM sodium nitrate. Cultures were shaken and supplemented with 1% CO_2_. Cultures naturally depleted of nitrogen after 6 days of growth. Sterile sampling was used for obtaining aliquots on a daily basis. Optical density at 750 nm (OD_750_) values were taken immediately after sampling. Samples for analysis were stored at 4°C until use. For cell cycle studies, triplicate *P. soloecismus* cultures were grown in 1 L volumes in 2.8 L spin flasks. Cultures were constantly bubbled with air and maintained at pH 8.25 by on-demand CO_2_ injection. Cultures were mixed by magnetic stirring at 200 rpm and illuminated with 800 μmolm^–2^s^–1^ in a 16 h/8 h light:dark cycle. Cultures were sampled every 2 h for 48 h for cell cycle assessment.

### Flow Cytometry Assessments (Cell Counts, FSC/SSC, DNA Ploidy, Lipid Accumulation)

Flow cytometry assessments were performed to determine cell concentration, relative cell size, DNA ploidy, and lipid accumulation in *P. soloecismus* as previously described (Unkefer et al., 2017; Steadman Tyler et al., 2019). Assessments were performed at the same time points and correlated to daily OD_750_ measurements 4 h into the light cycle. Unstained samples were used to determine cell concentration (cell/ml), relative size (FSC – forward scatter), and internal complexity (SSC – side scatter). Accumulation of neutral lipids was assessed using BODIPY 505/515 (D3921, Thermo Fisher Scientific, Waltham, MA, United States) staining and flow cytometry fluorescence assessment at selected time points during nitrogen replete, nitrogen starvation (*N* = 0), and nitrogen deplete culture conditions. For assessment of DNA content and replication, samples were taken every 2 h for 48 h, incubated with DyeCycle Orange (V35005, Thermo Fisher Scientific, Waltham, MA, United States), and assessed on the BD Accuri C6 Plus (BD Biosciences, San Jose, CA, United States) flow cytometer.

### DNA Methylation Inhibition

5-aza-2′-deoxycytidine was purchased from Sigma (A3656). 5AZA is preferable to 5-azacytidine for its retention in the cell; both exert proapoptotic effects (Gnyszka et al., 2013). 5AZA was prepared in 50% DMSO and 50% ice cold MilliQ water in the least possible volume for all final concentrations (0–80 μM) in 250 ml shaker flasks. Stock solutions of 5AZA were stored at −20°C; aliquots were thawed on ice prior to treatment to prevent drug instability and break down. Treatment occurred 4–5 h into the light cycle prior to DNA replication in *P. soloecismus* as determined by flow cytometry (see above) every day (days 1–16) of the growth cycle. The half-life of 5AZA in most mammalian cell cultures is between 8 and 10 h as determined in preclinical trials (Hollenbach et al., 2010).

### DNA Extraction

A modified, combined protocol was generated from the manufacturer’s instructions using E.Z.N.A. Plant DNA DS Mini Kit (D2411-01; Omega Bio-tek Inc., Norcross, GA, United States) and Quick-DNA Fungal/Bacterial Miniprep Kit (D6005; Zymo Research, Irvine, CA, United States) to isolate genomic DNA. Briefly, 400 μl of reconstituted cells were lysed using bead bashing lysis tubes and buffer at 4°C. Samples were treated with CSPL buffer and proteinase K solution and heated at 65°C for 30 min. Samples were centrifuged and cleared supernatant was passed through a mini column followed by RNase A treatment at RT. Cleared supernatant was treated with RBB Buffer and XP2 Buffer, vortexed, and transferred to a HiBind DNA Mini Column. HBC buffer and DNA wash buffers were added to the columns. Columns were allowed to air dry followed by 2 min incubation with elution buffer. DNA was purified using AMPure Purification Beads (100-265-900; PacBio, Menlo Park, CA, United States) in a 1:1 volumetric ratio per the manufacturer’s instructions. After separation on a magnetic rack and washing with 70% ethanol, the beads were incubated with PacBio elution buffer (101-633-500; PacBio, Menlo Park, CA, United States) for 10 min at RT. Purified DNA was removed in the supernatant and quantified using a Qubit dsDNA HS Kit (32854; Thermo Fisher Scientific). Lambda *Hin*dIII DNA marker was used to determine the DNA size (SM0101; Thermo Fisher Scientific). DNA integrity and size were assessed on E-Gel EX 1% agarose gel (G402001; Thermo Fisher Scientific).

### Global DNA Methylation Quantification

The presence of methylation on the 5′ carbon of cytosine in DNA was determined using the 5mC DNA ELISA Kit (D5325; Zymo Research, Irvine, CA, United States) per manufacturer’s instructions with minor changes. Modifications to the protocol included adding a 2.5% 5mC-DNA standard to the calibration curve, using 200 ng of input DNA, and quantification at 405 nm wavelength using a Tecan spectrophotometer (Tecan Life Sciences, Switzerland). For a positive control, *P. soloecismus* DNA was incubated with CpG Methylase (*M. Sssl*) and 12 mM of s-adenosyl methionine substrate (E2010; Zymo Research, Irvine, CA, United States) for 12 h at 30°C. The %5mC in DNA was determined using a saturation binding curve (non-linear fit) in GraphPad Prism 8 software (GraphPad, San Diego, CA, United States). Results are reported as %5mC.

### Whole Genome Bisulfite Sequencing (WGBS)

*Picochlorum soloecismus* samples were processed and analyzed using the Methyl-MaxiSeq library preparation, sequencing, and bioinformatics pipeline from Zymo Research (Irvine, CA, United States). Triplicate biological replicates from 5AZA treated and untreated cells over 5 days of the growth cycle representing replete and deplete nitrogen conditions were used for analysis. Briefly, Methyl-MaxiSeq libraries were prepared from 1 μg gDNA digested with two units of dsDNA Shearase^TM^ Plus (E2018-50; Zymo Research, Irvine, CA, United States). Fragments were end-blunted, the 3′-terminal-A extended, and purified using the DNA Clean & Concentrator Kit (D4003; Zymo Research, Irvine, CA, United States). A-tailed fragments were ligated to pre-annealed adapters containing 5mC instead of cytosine and adapter-ligated fragments were filled in. Fragments were treated with sodium bisulfite using the EZ DNA Methylation – Lightning Kit (D5030; Zymo Research, Irvine, CA, United States). Treated DNA was amplified with Illumina TruSeq indices; fragment DNA purity and size were confirmed on the Agilent 2200 TapeStation (Agilent Technologies, Santa Clara, CA, United States). DNA was sequenced using Illumina PE75 on the HiSeq (Illumina Inc., San Diego, CA, United States) instrument to 50X coverage.

### Methylation Alignment and Calling

Three biological replicates over 5 days of the growth cycle were sequenced for the presence of methylated cytosines. Sequencing reads from bisulfite-treated EpiQuest libraries were identified using standard Illumina base-calling software and then analyzed using bismark bowtie2^1^ for alignment. Methylation calling was performed using MethylDackel.^2^ Index files were constructed by bismark_genome_preparation command using the entire reference genome of *P. soloecismus* (GenBank PJAJ00000000). The –non-_directional parameter was applied while running bismark. All other parameters were set to default. For MethylDackel, parameters were also used to find sites in CHG and CHH contexts. All other parameters for MethylDackel were set to default. Methylation calls with greater than 20X coverage were validated against a list of all possible methylation sites in the genome. These validated sites were used to estimate global methylation profiles for each timepoint. All called sites are reported in Supplementary Tables (FigShare^3^). To obtain feature-length corrected methylation site frequencies in the genome, four features were used. These included “gene body,” “promoter,” “terminator,” and “intergenic regions.” Gene bodies denoted the protein coding regions and included introns and exons. Promoters and terminators were defined as the 500 bp 5′ and 3′ UTRs flanking gene bodies. Any sequence span not under these definitions of gene bodies, promoters, or terminators was marked as an intergenic region (IGR). These features are available as extended versions of the genomic annotation file published for *P. soloecismus* in the Supplementary Tables. Methylation sites were mapped to genomic features using Pandas (McKinney, 2010). Briefly, counts of called sites were obtained for each feature and divided by the size (bp) of that feature. The resulting site density value (in counts/bp) was multiplied by 1000 to express density as counts per kb. Variables (averages and standard deviations) were calculated with or without filtering out zero-count entries; data is reported without zero-count entries. The script for this calculation is available in GitHub: https://github.com/lanl/DNA_methylation_analysis. All raw fastq files, processed methylation tracks, and methylation calls are provided on the Gene Expression Omnibus (GEO) website under the accession record GSE155500.

### Differential Methylation Analysis

Data from Zymo Research included called sites, the number of total reads per site, and methylation ratio per site. The methylation ratio of each sampled cytosine is estimated as the number of reads reporting a cytosine divided by the total number of reads reporting a C or T [C/(C or T)]. Reads were culled according to NIH Roadmap Epigenomics Project (Bernstein et al., 2010). For the *P. soloecismus* genome (15.2 million base pairs, haploid), there was a median of 50X coverage for all sites. A Student’s *t*-test was performed for each cytosine with a minimum coverage of 20X aligned sequence reads (for every day in culture) to identify statistically significant methylation differences in each comparison. The differences in methylation ratios between Day 4 and Day 10 in culture (the first and last day of sequencing) were used to determine overall changes in methylation across the time course. All significant methylation ratio changes less than 0.1 and greater than −0.1 were not considered in the analysis. The same parameters for calling were used for sequences from 5AZA treated samples. To determine the effect of 5AZA on methylation ratios per site, differences in methylation ratios were calculated for each day in culture between treated and untreated cultures. Data is plotted as methylation ratio per day in culture.

### Methylation Visualization, Annotation, and Gene Cluster Analysis

To determine specific genes of interest that may contain sites of methylation, genomic annotations were added to sites with the most significant changes in methylation ratios (hyper or hypomethylation) from the LANL Greenhouse database.^4^ Open reading frames (ORF) extracted based on these annotations were assigned KEGG Orthologies (KOs) (Kanehisa et al., 2016a,b) using KofamKOALA (Aramaki et al., 2020), with an *E*-value cutoff of 1E-24. For each predicted ORF encoded in the annotations, we retained the KO assignment with the lowest *E*-value. LANL in-house software was used to map KOs to KEGG pathways (Kanehisa et al., 2016a,b), to determine if genes with significantly different methylation ratios over the cultivation time course clustered into particular metabolic processes. To visualize sites in a gene (multiple sites per gene) under two different conditions (either day in culture or 5AZA treatment), the relevant methylation sites were added to the annotations extant in the greenhouse database and color coded in Microsoft Excel for Mac 2019. Separate, augmented annotation files were created for each timepoint and condition to enable simultaneous viewing in standard genome browsers capable of interpreting the GFF3 format. All scripts used for data analysis and methylation calling are provided in GitHub.^5^

### Statistics

All statistical analyses were performed using GraphPad Prism8 software packages (version 8.4.1 (460), GraphPad Software, San Diego, CA, United States) with default parameters except when Bonferroni or Tukey’s *post hoc* analyses were performed. One-way ANOVA repeated measures was performed to determine methylation ratio differences of *P. soloecismus* across the growth cycle. Two-way ANOVA repeated measures analysis was used to compare 5AZA treated and untreated *P. soloecismus* phenotypes over the time course for three biological replicate cultures. These phenotypes included optical density, cell counts, cell size (FSC), cell complexity (SSC), and lipid accumulation. A Student’s *t*-test was used to evaluate %5mC and to evaluate the difference in methylation ratio between specific days in culture for treated or untreated cultures.

## Results

### The Presence of Epigenetic Machinery for DNA Methylation in *P. soloecismus*

**FIGURE 1:**
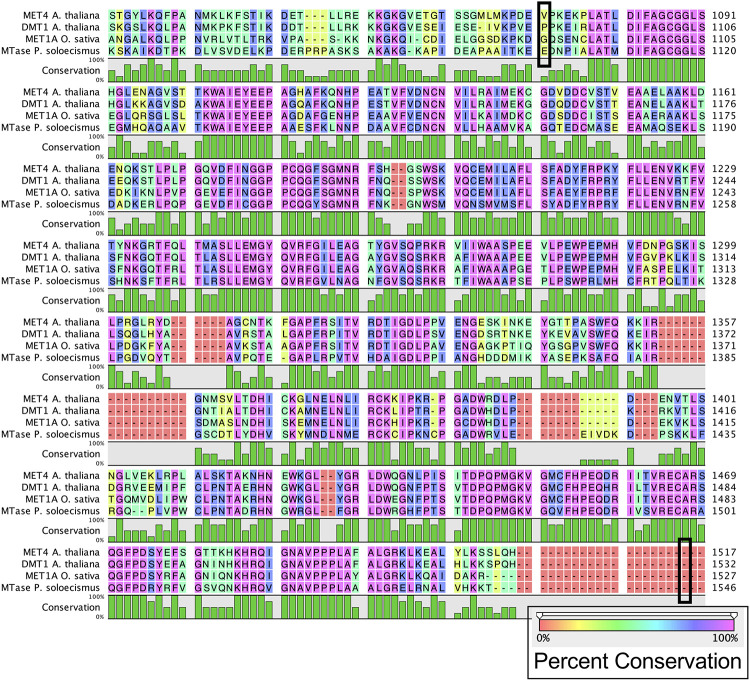
Alignment of common DNA methyltransferase enzymes with a candidate sequence from *P. soloecismus.* Amino acid sequences for DNA methyltransferase proteins MET4 (*A. thaliana*), DMT1 protein (*A. thaliana*), and MET1A protein (*O. sativa*) are aligned with a candidate sequence (NSC_00652) from *P. soloecismus*. All three enzymes have a C-5 cytosine methyltransferase domain (IPR001525). Alignment for this domain for MET4 is outlined (black box) from amino acid 1078 to amino acid 1512. The IPR001525 domain extends from amino acids 1093 to 1527 for DMT1 and 1092 to 1526 for MET1A (not outlined). The conservation of the amino acid sequences is shown on a colored scale with pink indicating the highest percent conservation. Below the amino acid sequence, conservation is quantified in green, with the tallest bars indicating 100% conservation. The protein sequence annotated with IPR001525 from *P. soloecismus* is shown and is a likely candidate for a cytosine methyltransferase (MTase).

Prior to experimental determination of DNA methylation, we interrogated the *P. soloecismus* genome for signatures of epigenetic machinery. In plants, several enzymes are responsible for imparting DNA modifications. Each enzyme has a specific function for methylation in a particular cytosine context (CpG, CHG, or CHH). DNA methyltransferase enzymes contain specific DNA binding domains in addition to their methyltransferase enzymatic activity domains. We found homologs for a number of enzymes involved in DNA methylation in the *P. soloecismus* genome, suggesting the possibility of DNA methylation in multiple contexts (Table 1A). Some of these enzymes have domains for both DNA binding and 5mC methyltransferase activity. These domains can be found in several different databases. Pfam is a curated database of expertly built multiple sequence alignments representing clusters of proteins and/or protein domains (Finn et al., 2015). Clusters of sequences are organized into “families,” and families are grouped at a higher level into “clans.” InterPro is a similar but broader database that combines information from member databases like Pfam, including CATH-Gene3D, TIGRFAMs, and PROSITE among others (Haft et al., 2003; Sigrist et al., 2012; Lewis et al., 2017; Sillitoe et al., 2018; Mitchell et al., 2019). These databases are particularly useful in annotation of remote homologs of proteins that may be found in newly annotated genomes. Both are commonly used in unison by automated annotation pipelines such as MAKER and AUGUSTUS (Stanke and Morgenstern, 2005; Cantarel et al., 2008). Interrogation of the *P. soloecismus* genome with InterPro and Pfam domains of interest (described in “Materials and Methods”) produced 14 hits for possible methyltransferase enzymes (Table 1B). This information was cross referenced with the homologs from Table 1A. Two of these potential enzymes were aligned with DNA methyltransferase enzymes from other species, demonstrating sequence variation except in important catalytic domains required for DNA methylation activity (Figure 1). This *in situ* data suggested that *P. soloecismus* contains at least two enzymes capable of covalent modification of DNA on the 5′ carbon of cytosine.

**TABLE 1A:** Top gene ID hits for homologs of DNA methyltransferases in *P. soloecismus.*

ID	Name	Domains
NSC_03941	s-adenosyl-methyltransferase	IPR002903, IPR023397
NSC_03950	Conserved hypothetical	PF13578
NSC_00652	Cytosine-5 DNA methyltransferase	IPR001525, IPR017198, IPR018117, IPR022702
NSC_01519	DNA-cytosine methyltransferase	IPR001525
NSC_00143	Hypothetical protein	IPR001025, IPR001357
NSC_06005	Meiosis expressed	IPR001025
NSC_00846	es43 protein	PR001025, IPR001965, IPR011011, IPR013083, IPR019786, IPR019787
NSC_03065	Chromodomain-helicase-DNA-binding protein	IPR000330, IPR000953, IPR001650, IPR014001, IPR016197, IPR023780
NSC_05938	Ankyrin repeat domain	IPR000953, IPR002110, IPR016197, IPR020683, IPR023780
NSC_03492	Elongation factor ef-3	IPR003439, IPR003593, IPR011989, IPR015688, IPR016024, IPR017871, IPR021133, IPR023780
NSC_00815	Arid bright DNA binding domain protein	IPR001487, IPR001606, IPR022702

*The IPR domains associated with genes of interest (Name and ID) in the *P. soloecismus* genome are provided.*

**TABLE 1B:** Epigenetic machinery domains of interest for 5mC DNA methylation and hits within the *P. soloecismus* genome.

Domain	Name	Hits	Function
IPR001025	BAH_dom	3	Protein-protein interaction module specialized in gene silencing
IPR001091	RM_Methylase	0	Site-specific DNA-methyltransferase, N-6 adenine-specific DNA methylase and cytosine-N4-specific
IPR001525	C5_MeTfrase	2	Methylates the C-5 carbon of cytosines in DNA
IPR002941	DNA_methylase_N4/N6	2	Family contains both N-4 cytosine-specific DNA methylases and N-6 Adenine-specific DNA methylases
IPR015270	RDM1_plant	0	Small protein that binds single-stranded methylated DNA; co-localizes with RNA polymerase II, AGO4 and DRM2 in the nucleus
IPR017198	DNMT1-like	1	Methylates CpG residues with a preference for hemimethylated DNA
IPR017985	MeTrfase_CN4_CS	0	Methylates the amino group at the C-4 position of cytosines in DNA
IPR018117	C5_DNA_meth_AS	1	Methylates the C-5 carbon of cytosines in DNA
IPR022702	Cytosine_MeTrfase1_RFD	2	Part of DNA (cytosine-5)-methyltransferase 1 that targets the protein towards replication foci
IPR023780	Chromo_domain	3	Conserved region of around 60 amino acids; condenses morphology of heterochromatin
IPR025794	Hist-Lys_N-MeTrfase_plant	0	Silencing mechanism; interacts with DNA CpNpG methylation requires the targeting of chromomethylase CMT3 to methylated histone
IPR029063	SAM-dependent_MTases	0	Transfer a methyl group from a donor (S-adenosyl methionine) to an acceptor
IPR030380	SAM_MeTfrase_DRM	0	Domains Rearranged Methylases (DRM1 and DRM2) are *de novo* cytosine methyltransferases from plants involved in the initial methylation of unmethylated DNA sequences
IPR030486	DNMT3L	0	Inactive regulatory factor of *de novo* DNA methyltransferases DNMT3A and DNMT3AB
IPR030487	C5_MeTfrase	0	Propagates methylation patterns with DNMT3B stimulating DNMT3A activity by promoting its association with nucleosomes
IPR030488	DNMT3B_ADD	0	ADD domain of DNMT3B
IPR033375	Cggbp1	0	A repetitive DNA-binding transcription regulator with target sites at CpG-rich sequences such as CGG repeats and Alu-SINEs and L1-LINEs
IPR036319	RDM1_sf	0	Superfamily includes protein RDM1 from *Arabidopsis thaliana*
IPR040175	TET1/2/3	0	Converts 5-methylcytosine to 5-hydroxymethylcytosine
PF00145	DNA methylase	0	Methylates the C-5 carbon of cytosines in DNA
PF00385	Chromo	0	Conserved region of 60 amino acids; condenses morphology of heterochromatin
PF01426	BAH	0	Protein-protein interaction module specialized in gene silencing; commonly found in chromatin-associated proteins including eukaryotic DNA (cytosine-5) methyltransferases and recognition complex 1 (Orc1) proteins
PF02182	SAD_SRA	0	Binds hemi-methylated CpG dinucleotides and other 5mC containing dinucleotides
PF09187	RDM1_plant	0	Family of plant proteins includes RDM1 from *Arabidopsis thaliana*; a component of the RNA-directed DNA methylation (RdDM) effector complex
PS51058	ZF_CXXC	0	Binds specifically to non-methylated CpG DNA; sequence found in mammalian DNMT1, MBD1, and MLL1

*IPR domain names and numbers involved in DNA methylation are listed; the number of hits per IPR domain found in the *P. soloecismus* genome is provided along with the function of the domain.*

### DNA Methylation Characteristics of *P. soloecismus*

DNA methylation was determined using two methods: 5mC ELISA and whole genome bisulfite sequencing (WGBS). Using the 5mC ELISA, 0.82% 5mC was detected in *P. soloecismus* gDNA. To generate a positive control, *P. soloecismus* gDNA was treated with CpG Methylase (*M. Sssl*). This positive control had 1.3% 5mC methylation (Supplementary Figure 1, *p* < 0.0001). This initial assessment of global 5mC suggested that genomic DNA methylation of *P. soloecismus* was low but amenable to alteration (based on treatment with the *M. Sssl* CpG methylase). Of note, the antibody-based ELISA from Zymo Research has a detection limit of >0.5% 5mC per 100 ng DNA.

**FIGURE 2:**
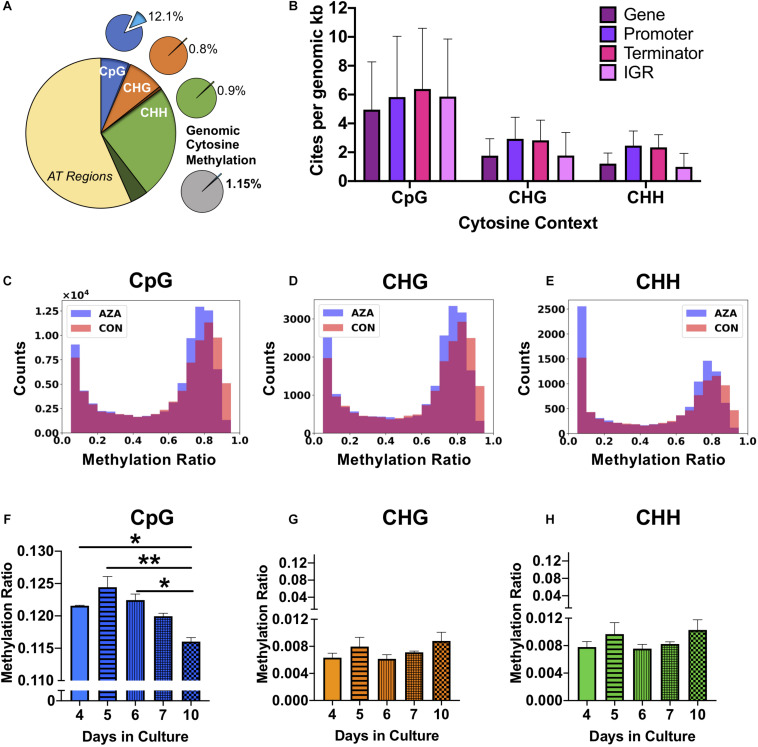
Genomic DNA methylation characteristics of *P. soloecismus*. **(A)** Graphical representation of genomic cytosine methylation in *P. soloecismus.* The larger circle depicts the *P. soloecismus* genome of 15.6 MB nucleic acids with AT content of 66% and GC content of 44%. The total number of CpG sites is shown in dark blue (1,014,486 sites) with called sites shown in lighter blue and labeled as CpG (94,195,597 sites). This constitutes approximately 93% of total CpG sites in the genome. The same quantification is presented for CHG (in orange) and CHH (in green), where 93% and 87% of sites were called, respectively. Of those called sites for CpG, the average methylation ratio is 0.121 or 12.1% (light blue slice of smaller pie). The average methylation ratio for CHG is 0.08% (orange) and for CHH is 0.09% (green). The gray circle depicts the sum total of 1.15% cytosine methylation with the majority derived from the CpG context (blue). **(B)** 5mC DNA methylation can be found in four features of the *P. soloecismus* genome: gene bodies, promoters, terminators, or intergenic regions (IGR). The number of sites per genomic kb for each of these four features in all three cytosine contexts is shown. Data is presented as mean ± SD (standard deviation). Representative histograms showing the distribution of 5mC DNA methylation in *P. soloecismus* for **(C)** CpG, **(D)** CHG, and **(E)** CHH cytosine contexts. Control samples are shown in orange, 5AZA samples are shown in purple, and the overlap is shown in magenta. The number of sites for each methylation ratio is shown in 0.1 bins. Given the low percentage of genomic methylation for *P. soloecismus*, sites from bin 0 to 0.05 were removed as most cytosines are unmethylated. **(F)** Global methylation ratios for CpG sites across the growth cycle of *P. soloecismus* as determined by WGBS. Tukey *post hoc* correction was performed for Student’s *t*-tests; the significance of those *post hoc* assessments is shown between days 4, 5, and 6 compared with day 10 in culture. **(G)** Global methylation ratios for CHG sites and **(H)** CHH sites across the growth cycle of *P. soloecismus* as determined by WGBS. No significant differences in global methylation across the growth cycle for CHG and CHH sites were found. Data are presented as mean ± SEM (standard error of the mean). * *p* < 0.05 and ** *p* < 0.01.

Whole genome bisulfite sequencing provides metrics for global and site-specific DNA 5mC methylation, including sequencing metrics and calls for methylation (Table 2). For the 15.2 MB *P. soloecismus* genome, cytosine content should have been called for approximately 1,014,486 CpG sites, 1,316,811 CHG sites, and 4,430,371 CHH sites (or about 44% of the genome). Approximately 93% of CpG and CHG sites and 87% of CHH sites were called for WGBS (Table 2). The methylation fraction for each sample was determined for each context to provide a picture of global methylation. For example, for Day 4 Control 1, there were 944,940 called CpG sites with an approximate methylation ratio of 0.123. Thus, approximately 12.3% of these sites had methylation or, as noted later, most CpG sites from this day in culture had approximately 12.3% methylation based on read counts. Methylation ratios were calculated as the number of methylated reads from the bisulfite converted sequences divided by the total number of reads for that particular site (# methylated C reads/# total C + T reads). From this assessment, we determined that on average, methylation occurred in 12.1% of CpG contexts, 0.8% of CHG contexts, and 0.9% of CHH contexts (Figure 2A). From a genome-wide perspective, the *P. soloecismus* genome had approximately 1.15% cytosine methylation (Figure 2A). This was determined by calculating the number of sites with methylation divided by the total genome size and normalized based on the number of called sites for the sequencing run. This methylation was divided across all cytosine contexts, with the majority of methylation occurring at CpG sites.

**TABLE 2:** Metrics for global and site-specific DNA 5mC methylation from whole genome bisulfite sequencing.

	Sequencing metrics				Median coverage of all sites			Called sites			Methylation fraction		
				
	Read pairs	Mapping efficiency	Unique CpGs	Coverage	CpG	CHG	CHH	CpG	CHG	CHH	CpG	CHG	CHH
Day 4 Control 1	41,023,876	75%	972,932	73	62	57	40	944940	1228396	3846923	0.123	0.008	0.009
Day 4 Control 2	33,648,831	77%	973,618	63	81	78	59	942047	1223285	3817927	0.122	0.006	0.007
Day 4 Control 3	29,579,086	76%	973,104	53	44	41	28	928325	1202508	3674044	0.121	0.005	0.007
Day 5 Control 1	25,654,725	77%	973,344	54	47	45	33	937428	1219080	3780033	0.123	0.008	0.010
Day 5 Control 2	29,937,985	79%	971,660	54	44	40	26	910613	1176724	3483664	0.126	0.005	0.007
Day 5 Control 3	45,111,988	77%	975,224	92	52	48	33	964233	1257465	4098185	0.123	0.010	0.013
Day 6 Control 1	36,639,600	80%	973,029	69	58	54	37	938805	1219379	3782740	0.123	0.005	0.007
Day 6 Control 2	40,794,105	74%	972,768	74	63	59	41	943226	1226052	3830516	0.122	0.007	0.009
Day 6 Control 3	29,191,188	74%	972,574	52	43	40	26	916792	1186033	3549572	0.123	0.006	0.007
Day 7 Control 1	33,885,815	77%	972,102	61	52	47	33	934725	1212452	3731159	0.123	0.007	0.009
Day 7 Control 2	40,610,069	72%	972,679	66	54	49	35	941356	1221066	3827818	0.120	0.007	0.008
Day 7 Control 3	26,226,738	77%	972,111	50	44	41	30	931596	1209022	3711732	0.119	0.007	0.008
Day 10 Control 1	44,523,335	72%	972,336	77	65	60	41	942568	1224561	3816369	0.119	0.008	0.009
Day 10 Control 2	31,106,461	73%	972,473	55	48	44	32	937025	1216470	3783515	0.115	0.008	0.009
Day 10 Control 3	31,278,928	71%	976,178	63	57	56	45	965823	1260295	4140910	0.117	0.011	0.013
Day 4 AZA 1	35,175,861	78%	974,284	68	58	54	39	953024	1240912	3971269	0.121	0.005	0.007
Day 4 AZA 2	25,810,214	80%	979,296	72	68	67	62	972892	1269534	4252504	0.119	0.017	0.018
Day 4 AZA 3	51,025,989	81%	973,858	103	53	49	34	956954	1247256	3995715	0.124	0.009	0.011
Day 5 AZA 1	23,711,109	77%	974,016	50	44	42	31	934472	1215456	3753189	0.122	0.009	0.011
Day 5 AZA 2	38,010,117	80%	976,674	88	81	79	65	970660	1266406	4206109	0.119	0.010	0.012
Day 5 AZA 3	24,571,651	78%	972,998	59	49	46	33	927143	1204554	3706979	0.129	0.009	0.010
Day 6 AZA 1	32,609,015	78%	973,530	62	46	43	29	935134	1213880	3753222	0.121	0.005	0.007
Day 6 AZA 2	36,869,188	78%	974,719	75	69	66	51	963066	1255704	4086914	0.119	0.009	0.010
Day 6 AZA 3	26,727,193	80%	974,765	59	53	51	40	955764	1245567	3991086	0.122	0.011	0.014
Day 7 AZA 1	37,076,497	78%	972,230	68	48	44	30	939065	1218808	3778410	0.120	0.007	0.008
Day 7 AZA 2	27,862,914	81%	973,613	54	46	43	29	934476	1212377	3747479	0.118	0.005	0.006
Day 7 AZA 3	32,739,443	80%	973,978	64	54	50	35	945266	1229569	3875733	0.120	0.005	0.007
Day 10 AZA 1	32,085,678	77%	974,843	77	66	63	46	950340	1237985	3940037	0.124	0.009	0.011
Day 10 AZA 2	31,462,613	79%	973,459	58	88	84	58	931975	1208968	3716882	0.121	0.006	0.007
Day 10 AZA 3	25,019,781	80%	972,153	47	39	36	24	908946	1173978	3481932	0.119	0.005	0.006

*WGBS was performed for *P. soloecismus* untreated (control) and treated (5-aza-2′-deoxycytidine, 5AZA) cultures over five days in triplicate. The read pairs, mapping efficiency, unique CpGs identified, and sequencing coverage per sample are provided. The median coverage per cytosine context is provided, along with the number of called sites and the average methylation fraction per context, per sample, per day in culture.*

For each cytosine context, we determined the relative abundance of DNA methylation in four genomic features: gene bodies, promoters, terminators, and intergenic regions. For CpG sites, there were, on average, 4.95 sites per kb for gene bodies, 5.83 sites per kb for promoters, 6.39 sites per kb for terminators, and 5.86 sites per kb for intergenic regions. Given that gene bodies are larger than most other features, there were more CpG sites of methylation found in genes; however, per kb, terminators had the most CpG sites (Figure 2B). The distribution of methylation in all contexts is bimodal (Figures 2C–E). Of the 699,653 called CpG sites on Day 4 in the first control sample (Supplementary Table 1), approximately 7% (48667) sites were largely methylated (>0.8 methylation ratio), and approximately 83% of sites were largely unmethylated (<0.2 methylation ratio). Approximately 2% of sites had moderate methylation (0.4–0.6). This finding correlated with the global methylation analysis indicating that 12.3% of CpG called sites for Day 4 had some methylation. Of that 12.3%, most sites were largely methylated (Figure 2C). Validation of called CpG sites resulted in other called CHG and CHH sites, which showed a bimodal distribution of methylation as well. Thus, while there were very few methylated CHG and CHH sites, the degree of methylation at those sites was large.

Changes in global and site-specific DNA methylation of *P. soloecismus* across its growth cycle were determined from the WGBS data. Global CpG DNA methylation decreased (hypomethylation) across the growth cycle (Figure 2F), with significant differences between early days (nitrogen replete) in the time course (Days 4, 5, 6) and late (nitrogen deplete) in the time course (Day 10) (*p* < 0.05). No significant changes in global DNA methylation in the CHG and CHH contexts across the growth cycle were observed (Figures 2G,H). All Supplementary Tables can be found on FigShare (see text footnote 3).

### Site Specific DNA Methylation Characteristics of *P. soloecismus*

To determine site specific hyper or hypomethylation across the time course, WGBS data was trimmed according to significant differences (*p* < 0.01) between Day 4 and Day 10 in culture for control cultures. Methylation differences between −0.1 and 0.1 were not considered in this analysis. Called sites were validated for cytosine context, some of which were CHH and CHG sites and removed from the analysis. There were 1102 significantly hypomethylated sites from Day 4 to Day 10 in culture with methylation differences ranging from −0.36 > x > −0.1 (Supplementary Table 2). There were 41 significantly hypermethylated sites from Day 4 to Day 10 in culture with methylation differences ranging from 0.19 > x > 0.1 (Supplementary Table 3). These sites were annotated and assigned KEGG orthologies, which were in turn, mapped to KEGG Pathways to determine the most impacted metabolic processes (also shown in the tables) using LANL in house software (Kanehisa et al., 2016a).

**FIGURE 3:**
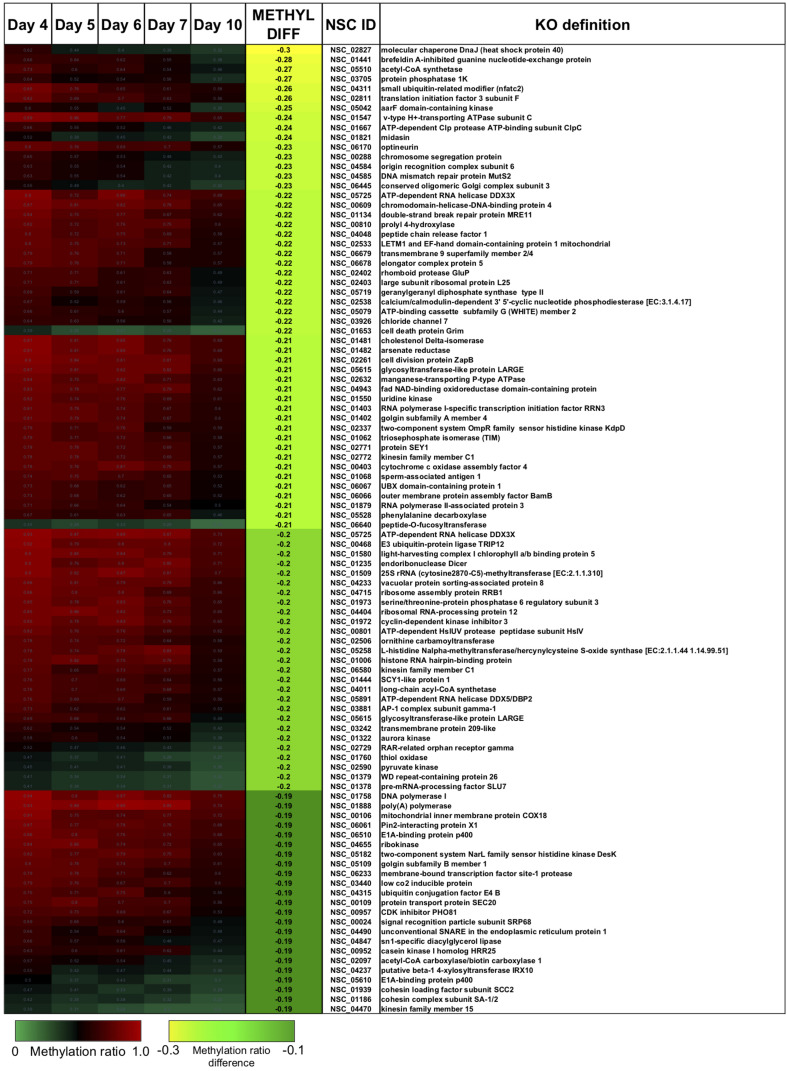
The top 100 CpG sites that became significantly hypomethylated across the growth cycle of *P. soloecismus.* The top 100 annotated sites with the greatest change in methylation ratio across the growth cycle from Day 4 to Day 10 are listed (*p* < 0.01 for all sites). The greatest change in methylation ratio (–0.3) is shown under the column METHYL DIFF in yellow; change in methylation ratio decreases in absolute value down the column (dark green). Sites are largely methylated (red) and become less methylated (darker, black). Few sites have lower methylation (lighter green) and become less methylated over time (darker green). Some sites start with moderate methylation (black) and become hypomethylated (green). Annotations (NSC_ID corresponding to the *P. soloecismus* genomic ID) and the KO (KEGG Orthologies, within E-24) definitions are provided.

**FIGURE 4:**
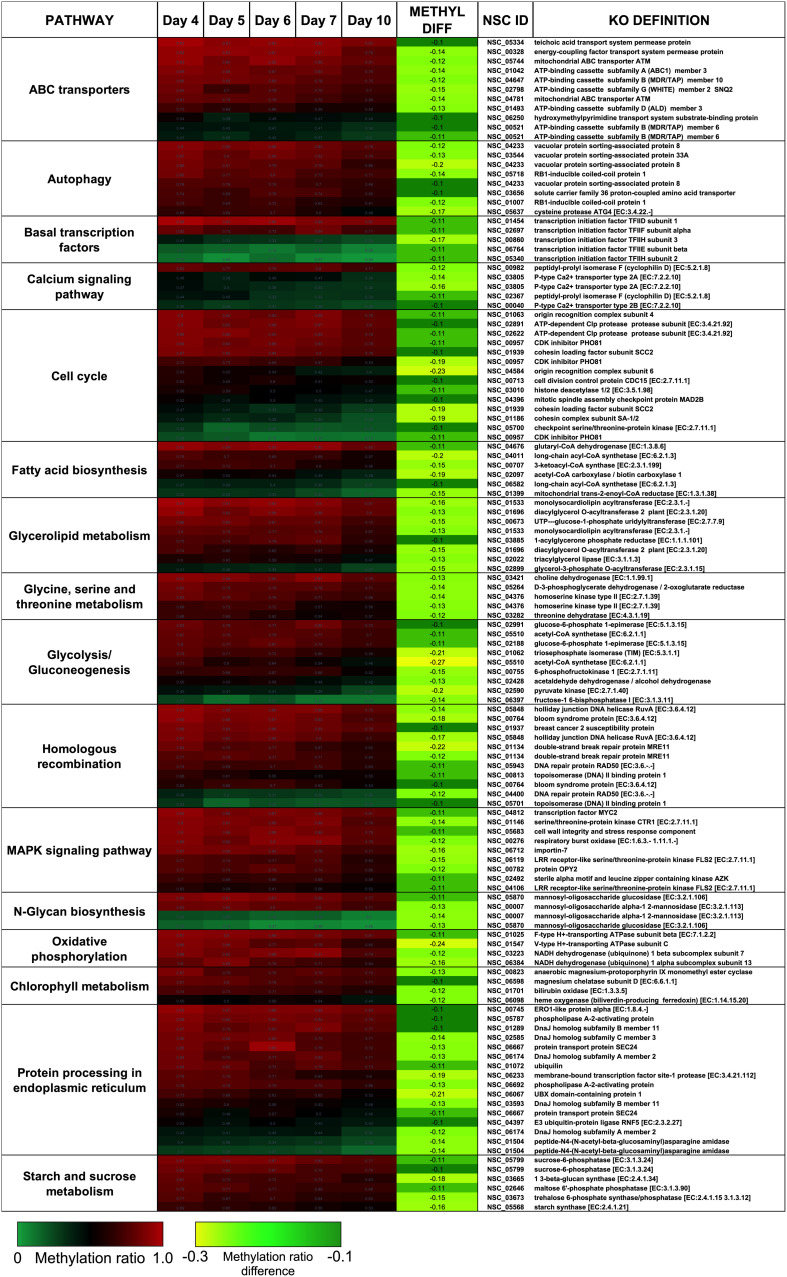
CpG sites that became hypomethylated across the growth cycle of *P. soloecismus* belong to metabolic pathways important for algal biofuel characteristics. CpG sites from the 1102 most significantly hypomethylated sites are shown; these were chosen based on their annotations to specific metabolic pathways, and their role in lipid accumulation and the cell cycle. All pathways have sites with decreased methylation ratios across the time course (labeled as METHYL DIFF, derived from subtracting Day 4 methylation ratio from Day 10, shown as negative values). All methylation differences are significant (*p* < 0.009). Annotations (NSC_ID corresponding to the *P. soloecismus* genomic ID) and the KO (KEGG Orthologies, within E-24) definitions are provided.

There were two main features of the sites that became hypomethylated across the *P. soloecismus* growth cycle. First, most sites were largely methylated (average methylation ratio was 0.72) and became hypomethylated but not completely demethylated (average methylation ratio was 0.58 by Day 10 in culture). Very few sites started with low methylation and became even less methylated, though there were some sites with moderate methylation that became hypomethylated. This pattern can be seen in the top 100 sites with the greatest change in methylation ratio (Figure 3, *p* < 0.01). The third most significantly hypomethylated site was annotated as acetyl CoA synthetase, an important protein involved in lipid synthesis (Figure 3). The 1102 significantly hypomethylated sites were mapped to specific metabolic pathways deemed important for algal biofuel species (Figure 4). Of note, several sites aligned with genes involved in the cell cycle, fatty acid synthesis, amino acid metabolism, glycolysis, gluconeogenesis, MAPK signaling, and photosynthesis. Other significantly hypomethylated sites were annotated to genes involved in ribosome formation, RNA synthesis, splicing, transport, and degradation (Supplementary Table 4). All Supplementary Tables can be found on FigShare (see text footnote 3).

### DNA Replication in *P. soloecismus*

**FIGURE 5:**
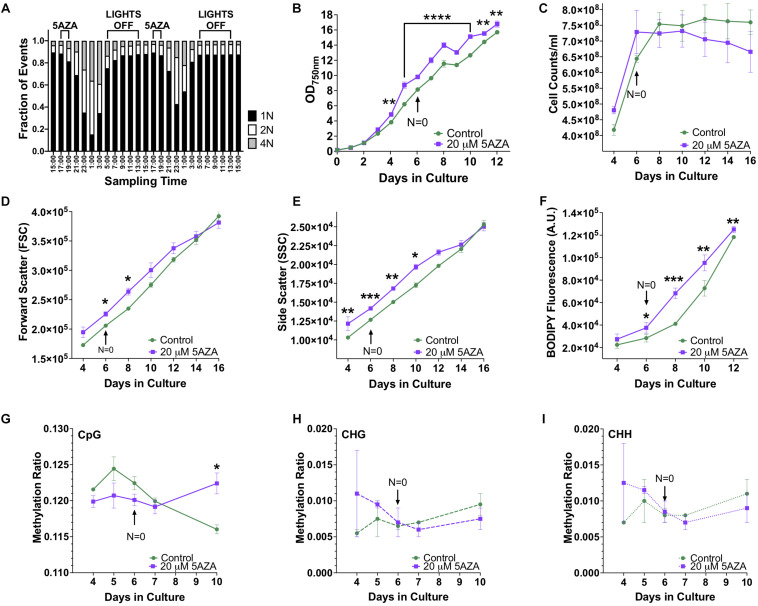
The effect of 5AZA on cellular characteristics, biomolecule composition, and global DNA methylation of *P. soloecismus*. **(A)** DNA ploidy was determined using flow cytometry assessment of DyeCycle Orange fluorescence over 24 h. The histogram depicts quantitative assessment as measured by the number of cells with DCO fluorescence (fraction of events) in the appropriate gates (*N* = 1, 2, 4) every 2 h over a 24 h period. N indicates relative ploidy, where *N* = 1 is haploid, *N* = 2 is diploid, etc. DCO intensity was measured in duplicate and data are shown as mean ± SD (standard deviation). The 16:8 light/dark cycle is indicated on the graph: lights were turned off at 5:00 AM and on at 13:00 (1:00 PM). The maximum number of cells in haploid state before DNA replication is between 17:00 and 19:00 and thus indicates the appropriate time for 5AZA treatment indicated on graph. **(B)** *P. soloecismus* shaker cultures were treated daily 4–5 h into the light cycle with 20 μM 5-aza-2′deoxycycdine (5AZA). Optical density (OD_750nm_) was assessed to track growth (prior to 5AZA treatment each day). Treatment with 20 μM 5AZA significantly altered the optical density of *P. soloecismus* after 4 days of treatment; this effect is perpetuated throughout the entire time course (*p* < 0.0001). Bonferroni *post hoc* correction was performed for Student’s *t*-tests; the significance of those *post hoc* assessments is shown on the graph. **(C)** Cell counts were determined for Days 4–16 in culture in control and 20 μM 5AZA treated *P. soloecismus* cultures; there was no significant effect of 5AZA treatment. **(D)** Forward scatter (FSC) was determined for Days 4–16 in culture in control and 20 μM 5AZA treated *P. soloecismus* cultures; an initial effect of 5AZA treatment was observed but not propagated throughout the time course. Significant Bonferroni corrected *post hoc* analyses are shown on the graph with asterisks for Days 6 and 8 in culture. **(E)** Side scatter (SSC) was determined for Days 4–16 in culture in control and 20 μM 5AZA treated *P. soloecismus* cultures; a significant effect of 5AZA treatment on the complexity of cells across most of the time course was determined (*p* < 0.05). Significant Bonferroni corrected *post hoc* analyses are shown on the graph with asterisks. **(F)** Lipid accumulation (as determined by BODIPY fluorescence) was determined for Days 4–12 in culture in control and 20 μM 5AZA treated *P. soloecismus* cultures. Treatment with 5AZA after 6 days induced significant lipid accumulation in *P. soloecismus*, an effect that was perpetuated across the time course (*p* < 0.0001). Bonferroni *post hoc* correction was performed for Student’s *t*-tests; the significance of those *post hoc* assessments is shown on the graph with asterisks for Days 6, 8, 10, and 12 in culture. **(G)** Global DNA methylation was determined for Days 4–10 in culture in control and 20 μM 5AZA treated *P. soloecismus* cultures; methylation ratios for CpG methylation across days in culture show no effect of 5AZA until Day 10 in culture (*p* < 0.05). **(H)** Methylation ratios for CHG and **(I)** CHH sites across days in culture show no effect of 5AZA. For all graphs, *N* = 0 denotes nitrogen starvation in the culture, and data are presented as mean ± SEM (standard error of the mean). * *p* < 0.05; ** *p* < 0.01; *** *p* < 0.001; and **** *p* < 0.0001.

Modifications to the epigenome of an organism can be induced by altering the expression and function of epigenetic machinery within the cell using drugs such as 5AZA. To determine the optimal time of drug delivery, DNA ploidy of *P. soloecismus* was assessed every 2 h over a 48 h period. As previously described, *P. soloecismus* has a haploid genome; DNA populations are denoted as *N* = 1, *N* = 2, and *N* = 4 in flow cytometry data (Gonzalez-Esquer et al., 2018; Steadman Tyler et al., 2019). The stable haploid population (*N* = 1) was present 3–5 h into the light cycle (Figure 5A). The *N* = 1 population increased during the “dark” part of the diurnal cycle and had the greatest number of cells from 17:00–19:00 h, or 4–6 h into the light cycle. As *N* = 1 population diminished, the *N* = 2 and *N* = 4 populations increased due to DNA replication. A large *N* = 4 population emerged 10 h into the light cycle (23:00). At 14 h into the light cycle (3:00) the cells started to divide. Cell counts and forward scatter (FSC, indicative of cell size) were also determined for these times points. From these experiments, we determined introduction of 5AZA prior to DNA replication would induce the most efficacious phenotype. Thus, drug treatment occurred 4–5 h into the light cycle (between 17:00 and 18:00).

### 5AZA Treatment Altered Growth of *P. soloecismus*

*Picochlorum soloecismus* was cultivated in shaker flasks and optical density measurements were taken approximately 4–5 h into the light phase of growth every 24 h for 16 days. Algae were treated with 0–20 μM 5AZA and the dose response was determined (Supplementary Figure 2). Drug treatment pharmacodynamics follow an inverted-U dose response with low and high concentrations of the same drug not eliciting a significant response (Tyler et al., 2018). We assessed 0–80 μM of 5AZA treatment and found this inverted -U dose response (data not shown); 20 μM 5AZA induced the most distinct growth response. The treatment effect of 20 μM 5AZA was repeated with biological triplicates and appropriate controls. Optical density at 750 nm (OD_750_) is an appropriate initial measurement of growth phenotype for *P. soloecismus.* We did not observe a decrease in OD_750_ in response to drug treatment as expected; in fact, treatment with 20 μM 5AZA increased the OD_750_ (Figure 5B, *p* < 0.0001). The effect of 5AZA treatment became apparent (and significant) after 4 days of treatment in culture. Statistical analysis suggested a significant main effect of time in culture and treatment with 5AZA with a significant interaction between the factors (*p* < 0.0001) (All ANOVA statistical analyses, including *F* and *p* values are provide in Table 3). Nitrogen starvation occurred on Day 6 of culture (data not shown) and may have had a combined effect with 5AZA treatment. It is typical of all algae cultures to utilize available nitrogen for rapid growth, and thus, “nitrogen starvation” occurs later in cultivation (Sharma et al., 2012; Banerjee et al., 2017). Overall, treatment with 20 μM 5AZA significantly altered the optical density of *P. soloecismus* after 4 days of treatment; this effect was perpetuated throughout the growth cycle (Figure 5B, *p* < 0.0001).

**TABLE 3:** F and *p*-values for ANOVA statistical analyses.

Assessment	Main effect of time in culture (Factor 1)	Main effect of 5AZA treatment (Factor 2)	Interaction between factors	*Post-hoc* Analyses
Effect of time on CpG methylation ratios (Figure 2F) *1-way ANOVA repeated measures*	*F*(4,5) = 11.98 *p* = 0.0089	No AZA treatment	N/A	Tukey corrected *post hoc*: Day 4 to Day 10: *p* < 0.05 Day 5 to Day 10: *p* < 0.01 Day 6 to Day 10: *p* < 0.05
Effect of 5AZA on optical density (Figure 5B) *2-way ANOVA repeated measures*	*F*(12,120) = 2868 *p* < 0.0001	*F*(1,10) = 34.88 *p* < 0.0001	*F*(12,120) = 19.88 *p* < 0.0001	Bonferroni corrected *post-hoc*: Days 4, 10, 11: *p* < 0.01 Days 5-10: *p* < 0.0001
Effect of 5AZA on cell counts (Figure 5C) *2-way ANOVA repeated measures*	*F*(6,48) = 45.25 *p* < 0.0001	*F*(1,9) = 0.02904 *ns*	*F*(6,48) = 2.778 *p* < 0.05	Bonferroni corrected *post-hoc*: *ns*
Effect of 5AZA on forward scatter (FSC) (Figure 5D) *2-way ANOVA repeated measures mixed effects*	*F*(2.482,24.21) = 431.1 *p* < 0.0001 Geisser-Greenhouse’s epsilon 0.4137	*F*(1,10) = 4.083 *ns*	*F*(6,59) = 3.721 *p* < 0.01	Bonferroni corrected *post-hoc*: Day 6, 8: *p* < 0.05
Effect of 5AZA on side scatter (SSC) (Figure 5E) *2-way ANOVA repeated measures mixed effects*	*F*(2.205,21.31) = 521.0 *p* < 0.0001 Geisser-Greenhouse’s epsilon 0.3675	*F*(1,10) = 9.694 *p* < 0.05	*F*(6,58) = 4.458 *p* < 0.001	Bonferroni corrected *post-hoc*: Days 4 and 8: *p* < 0.01 Day 5: *p* < 0.001 Day 10: *p* < 0.05
Effect of 5AZA on lipid accumulation (Figure 5F) *2-way ANOVA repeated measures*	*F*(1.138,11.38) = 784.5, *p* < 0.0001	*F*(1,10) = 3677 *p* < 0.0001	*F*(4, 40) = 12.49 *p* < 0.0001	Bonferroni corrected *post-hoc*: Day 6: *p* < 0.05 Day 8: *p* < 0.001 Days 10 and 12: *p* < 0.01

*All ANOVA statistical analyses for each type of assessment is provided. The F and *p*-values for the main effect of time in culture, the main effect of treatment in culture, the factors’ interaction, and any *post-hoc* analyses are listed.*

### 5AZA Treatment Altered Cellular Characteristics and Biomolecule Composition of *P. soloecismus*

Increased optical density of algae cells can result from a number of cellular and physiological changes. Cell counts, forward scatter (FSC, indicative of cell size), and side scatter (SSC, indicative of cell complexity) were assessed via flow cytometry (Figures 5C–E). Cell counts were not significantly impacted by 5ZA treatment (Figure 5C), though this lack of significance is likely due to the large variance in measurement. Both FSC and SSC were impacted by 5AZA treatment in similar ways: initially 5AZA significantly increased both FSC (Figure 5D, *p* < 0.01) and SSC (Figure 5E, *p* < 0.001) until Days 8 and 10, respectively, but this effect was abrogated as the days in culture increase. In other studies of microalgae cultivation, lack of change in cell counts accompanied by an increase optical density, FSC, and SSC suggests altered cellular composition particularly of biomolecules like neutral lipids (Bono et al., 2015; Gonzalez-Esquer et al., 2019; Steadman Tyler et al., 2019). Using a BODIPY fluorescent probe (Steadman Tyler et al., 2019), we found that lipid accumulation was significantly increased after 4 days of 5AZA treatment (Figure 5F). This increase remained apparent across the growth cycle of *P. soloecismus* (*p* < 0.0001). Every day in culture had significantly increased lipid accumulation in response to 5AZA (Figure 5F). While Day 4 showed a 22% increase in lipid accumulation, this was not significant as the coefficient of variance was 12.92 and 15.27 for the control and 5AZA treated cultures, respectively. This increased variance in measurement likely contributed to the lack of significance. Similarly, Day 12 showed a 5% increase in lipid accumulation, but the coefficient of variance was low for both control (0.72) and 5AZA treated cultures (1.98), providing statistical significance. 5AZA significantly increased lipid accumulation on Day 6 (32%), Day 8 (66%), and Day 10 (31%) all of which had nominal coefficients of variance (CoV < 1%) (All ANOVA statistical analyses, including *F* and *p* values are provide in Table 3).

### The Effect of 5AZA Treatment on DNA Methylation in *P. soloecismus*

Whole genome bisulfite sequencing was performed on samples treated with 5AZA across the time course (Days 4, 5, 6, 7, and 10 in culture). There was no change in total global methylation for any cytosine context (CpG, CHH, and CHG) in response to 5AZA treatment except on CpG sites on the last day assessed (Day 10, *p* < 0.05 for CpG) (Figures 5G–I). The methylation ratios across all called sites for each day in culture for all three replicates were averaged for these calculations.

**FIGURE 6:**
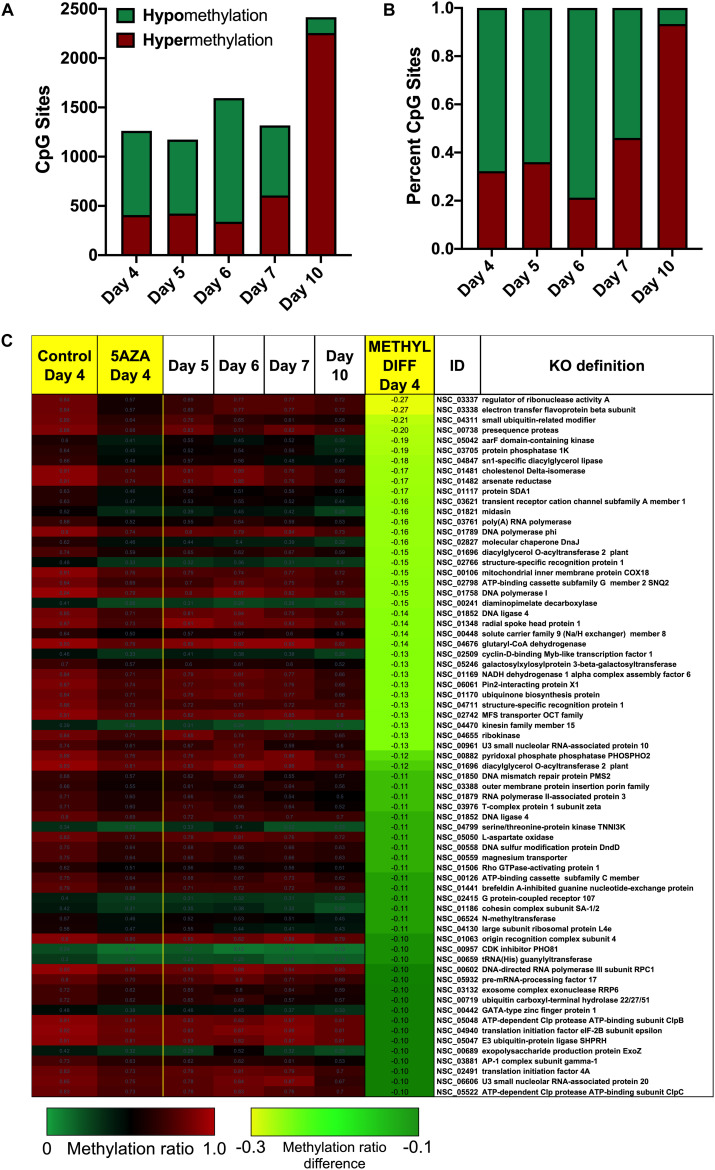
Sites-specific changes in CpG methylation ratios due to 5AZA treatment. **(A)** The number of CpG sites with significantly altered methylation ratios after 5AZA treatment (*p* < 0.05) per day in culture are shown. **(B)** The percent of hypomethylated or hypermethylated sites normalized to total sites affected by 5AZA per day are shown. Green, hypomethylation; Red, hypermethylation **(C)** CpG sites in *P. soloecismus* that are significantly hypomethylated on Day 4 of 5AZA treatment are shown. Methylation ratios from control and 5AZA treated cultures on Day 4 are side by side, followed by methylation ratios for control cultures for Days 5–10, all of which are significantly hypomethylated. Methylation differences between control and 5AZA treated cultures on Day 4 are labeled as METHYL DIFF Day 4; all methylation differences are significant (*p* < 0.05). Annotations (NSC_ID corresponding to the *P. soloecismus* genomic ID) and the KO (KEGG Orthologies, within E-24) definitions are provided.

**FIGURE 7:**
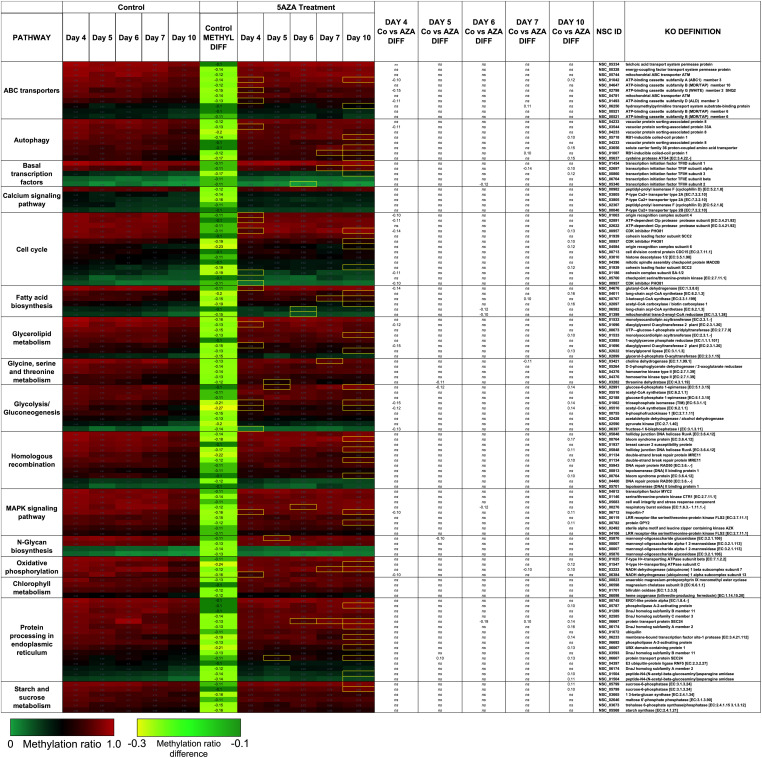
Hypomethylated CpG sites belonging to metabolic pathways important for algal biofuel characteristics are most impacted by 5AZA treatment on Day 10. CpG sites become hypomethylated across the growth cycle (in response to nitrogen starvation). Methylation differences across the growth cycle are labeled as METHYL DIFF. All methylation differences are significant (*p* < 0.009). Significant differences in methylation ratios (–0.1 > x > 0.1) due to 5AZA treatment are outlined in yellow boxes (*p* < 0.05). The *p*-values for methylation differences between control and 5AZA treated cultures for each site are labeled as “Day X Co vs. Aza DIFF.” Annotations (NSC_ID corresponding to the *P. soloecismus* genomic ID) and the KO (KEGG Orthologies, within E-24) definitions are provided.

Treatment with 5AZA did not impact the percent of global DNA methylation for the entire *P. soloecismus* genome. Yet, for specific sites, 5AZA treatment induced hypomethylation *and* hypermethylation (Figures 6A,B). Differences in methylation ratios were determined for each day comparing control versus 5AZA treated cultures. The most significant differences (*p* < 0.05) were kept, and sites with methylation ratio differences in the −0.1 < x < 0.1 range were trimmed from the analysis (as performed in all analyses). Called sites were validated for context and annotated (see methods). By Day 4 in culture, 855 sites were hypomethylated and 407 sites were hypermethylated in response to 5AZA treatment (Figure 6A). The most significant impact of 5AZA treatment occurred on Day 10 in culture: 2255 sites were hypermethylated and 161 sites were hypomethylated (Figure 6A). Given that mitosis does not occur after Day 6 in culture, this effect is likely due to the lack of efficacy of the 5AZA drug, which begins on Day 7 with 607 sites of hypermethylation. Days 4, 6, and 10 had the most significant effect of 5AZA coinciding with the most physiologically relevant days in culture (Figures 5F, 6A). All sites with significant methylation ratio differences (hyper and hypomethylation) are provided in Supplementary Tables 5–9 for all days in culture; these sites are also annotated. A subset of hypomethylated CpG sites (∼190) in response to nitrogen starvation had significantly differential methylation in response to 5AZA treatment on Day 4 of the time course (Figure 6C, *p* < 0.05). The methylation ratios for the following days in culture, starting on Day 5 for control cultures, were more similar (closer in color) to the 5AZA-treated culture on Day 4. Thus, 5AZA-induced hypomethylation early in treatment was similar to the hypomethylation that occurred across the growth cycle in response to nitrogen starvation (Figure 6C). This trend suggests that the 5AZA simply shifted the hypomethylation status of specific sites sooner than would normally occur during nitrogen starvation. For sites that were significantly hypomethylated during the growth cycle that were mapped to metabolic pathways deemed important for algal biofuel species (Figure 4), the pattern was not as clear. These sites were either not impacted by 5AZA or were hypomethylated early after 5AZA treatment (Day 4); many of these sites became hypermethylated after several days of 5AZA treatment (Day 10) (Figure 7).

**FIGURE 8:**
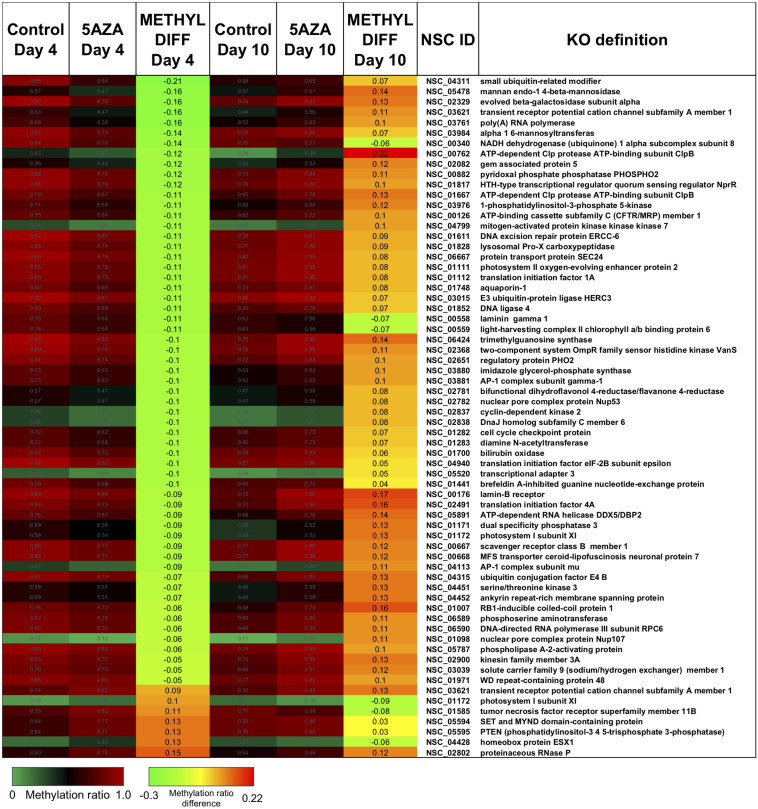
The top CpG sites with significantly altered methylation ratios due to 5AZA treatment on Day 4 and Day 10. Average methylation ratios are shown for control and 5AZA treated cultures on Day 4 and Day 10. All methylation ratio differences between control and 5AZA treated cultures, labeled as METHYL DIFF Day 4 or Day 10, are significant (*p* < 0.05). Annotations (NSC_ID corresponding to the *P. soloecismus* genomic ID) and the KO (KEGG Orthologies, within E-24) definitions are provided.

Interestingly, there was a subset of sites where 5AZA induced significant hypomethylation on Day 4 and significant changes in methylation ratio on Day 10 (Figure 8, *p* < 0.05). For this analysis, sites with significant methylation differences between −0.1 < x < 0.1 were not considered unless either Day 4 or Day 10 fulfilled the criteria for selection. All of these sites became significantly hypomethylated across the time course without 5AZA treatment due to nitrogen starvation. Of note, a pattern emerged of significant hypomethylation on Day 4 followed by hypermethylation of the same site by Day 10 (Figure 8). The genes associated with these sites did not fall into a particular category. Of the 855 CpG sites that became hypomethylated on Day 4 by 5AZA, 283 of these sites remained hypomethylated with no significant change by Day 10 (Supplementary Figure 3).

**FIGURE 9:**
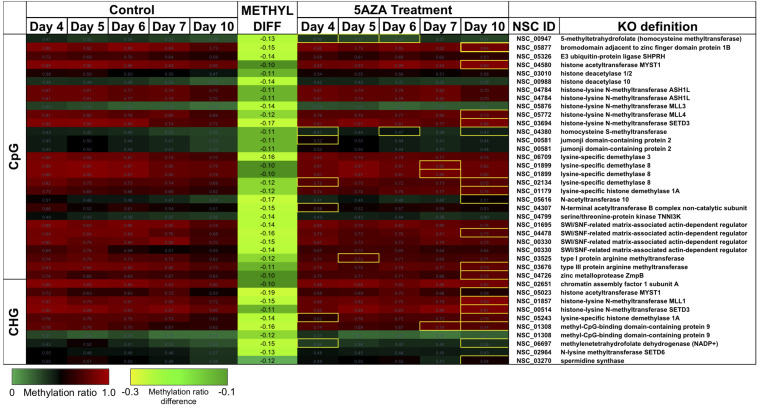
CpG and CHG sites in *P. soloecismus* belonging to genes for chromatin modifying proteins with differential methylation ratios. Methylation ratios across the growth cycle (control) and in response to 5AZA treatment are shown for sites annotated to epigenetic machinery. Significant hypomethylation across the growth cycle (for controls) occurs for all sites shown (labeled as METHYL DIFF) (*p* < 0.009). Methylation ratios significantly altered by 5AZA are outlined in yellow with the majority of differences (hypermethylation) occurring on Day 10 of 5AZA treatment. These are calculated by subtracting the methylation ratios for a specific day in the controls from the 5AZA treated methylation ratios (i.e., methyl ratio Day 4 5AZA – methyl ratio Day 4 control). Annotations (NSC_ID corresponding to the *P. soloecismus* genomic ID) and the KO (KEGG Orthologies, within E-24) definitions are provided.

There were several sites of cytosine methylation found within or near genes involved in epigenetic regulation. Some of these sites (CpG and CHG) became hypomethylated across the time course (Figure 9) and were impacted by 5AZA treatment; many of them became significantly hypermethylated in response to 5AZA treatment by Day 10 in culture. These sites included histone methyltransferases (MLL and SET proteins), histone acetyltransferases (MYST1), histone deacetylases (HDAC1/2), and chromatin remodeling proteins (SWI/SNF). To date, histone modifications have not been measured in *P. soloecismus*. However, this data suggests that this microalgae may use histone modifications for regulation and that these sites are themselves regulated by DNA methylation. All Supplementary Tables can be found on FigShare (see text footnote 3).

## Discussion

Approximately 40,000 species of microalgae have been reported, though some estimates are double (Khan et al., 2018). Many of these species have not been sequenced and even fewer have epigenome characterization. The handful of algal methylomes available do not show a distinctive pattern of DNA methylation; further, there is some disagreement on the amount and distribution of methylation within the same species (Hattman et al., 1978; Feng and Chiang, 1984; Cerutti, 1997; Wu-Scharf et al., 2000; Babinger et al., 2001, 2007; Jeong et al., 2002; Feng et al., 2010a; Zemach et al., 2010; Maumus et al., 2011; Veluchamy et al., 2014; Lopez et al., 2015). Collectively, the methylation context, location, and percentage all vary significantly (so far); this is likely due to the highly divergent nature of algal genomes.

Given the potential role of *P. soloecismus* in the future of algae-based biofuel production, understanding even one small aspect of its genomic regulation could have larger implications for the algae field (Barry et al., 2016; Unkefer et al., 2017; Gonzalez-Esquer et al., 2019). The three major findings of this work are as follows: (1) *P. soloecismus* has a small but quantifiable amount of global DNA methylation; (2) this methylation changes during the growth cycle of *P. soloecismus* in response to nitrogen starvation and 5AZA treatment, leading to the induction of lipids; and (3) CpG sites exhibit dynamic methylation in genes involved in fatty acid biosynthesis and the cell cycle. All three findings suggest that epigenetic regulation plays a key role in the growth and productivity of *P. soloecismu*s.

We determined the following features of DNA methylation in *P. soloecismus*. First, the *P. soloecismus* genome encodes for at least two putative DNA methyltransferases. Approximately 1.15% of the *P. soloecismus* 15.2 MB genome contains some form of cytosine methylation. Contextually, this methylation occurs in a bimodal distribution predominately in (∼12.1%) CpG sites, though there are some (<1%) CHH and CHG sites of methylation. Methylated sites are found in all genomic features, though terminators have the most abundant CpG sites per kilobase of the genome. For context, DNA methylation in microalgae varies from less than 1% CpG methylation in *C. reinhardtii* (Lopez et al., 2015) and *Volvox carteri* (Babinger et al., 2007) to almost 80% CpG methylation in *Chlorella variabilis* NC64A (Zemach et al., 2010).

We found that DNA methylation in *P. soloecismus* is dynamic and responsive to the environment. Treatment of *P. soloecismus* gDNA with a methylase derived from *Escherichia coli* increased global DNA methylation, suggesting sites of methylation are responsive to perturbation. Global hypomethylation on CpG sites occurred across the growth cycle of *P. soloecismus*, potentially in response to nitrogen starvation, with the greatest impact occurring by Day 10 in culture under severe nitrogen depletion conditions. We have previously observed that during nitrogen starvation, *P. soloecismus* ceases dividing and accumulates lipids in response to this stress. Several of the hypomethylated CpG sites are annotated as genes in pathways involved in lipid biosynthesis, including acetyl-CoA synthetase, long-chain acyl-CoA synthetase, 3-ketoacyl-CoA synthetase, acetyl-CoA carboxylase, and glutaryl-CoA dehydrogenase. Acyl-CoA synthetases have been shown to stimulate the release of lipids in *C. reinhardtii* (Jia et al., 2016), while acetyl-CoA production is associated with increased lipid accumulation in green algae (Avidan et al., 2015). The last step of lipid biosynthesis dependent on acyl-CoA is catalyzed by diacylglycerol acyltransferase (DGAT) (Wei et al., 2017); a CpG site within this gene (annotated as diacylglycerol O-acyltransferase 2) became hypomethylated across the growth cycle of *P. soloecismus* as well. Further, several CpG sites within genes involved in glycolysis and gluconeogenesis also became hypomethylated; the formation of glucose 6-phosphate eventually leads to the synthesis of pyruvate for fatty acid biosynthesis (Xue et al., 2017). This suggests that DNA methylation plays a role in nitrogen responses in *P. soloecismus* and potentially regulates genes that are involved in stress responses and lipid accumulation.

To determine how important DNA methylation is for the survival of *P. soloecismus*, we employed the use of 5AZA in culture. Once inside a cell, 5AZA forms a covalent bond with the DNA methyltransferase (DNMT) enzyme during DNA replication and inhibits the DNMT from binding to the newly synthesized DNA. Maintenance DNA methylation from hemimethylated DNA on the lagging strand is impeded by the presence of 5AZA. Over the growth cycle, daughter cells generated during mitosis lose DNA methylation (Stresemann and Lyko, 2008). Previous studies have demonstrated significant DNA demethylation and cellular responses (including apoptosis and DNA damage) after 5AZA treatment in several cell types (Christman, 2002; Madlung et al., 2002; Chang and Pikaard, 2005; Akimoto et al., 2007; Karahoca and Momparler, 2013). We anticipated that 5AZA would exert similar effects on *P. soloecismus*.

We did not observe global changes in DNA methylation in response to daily 20 μM 5AZA treatment: markedly, despite obvious differences in phenotype, it seemed that cytosine methylation was unaffected by the drug, except on Day 10 in culture when there was a striking increase in global methylation with drug treatment. Deeper analysis into site-specific changes in methylation ratios in response to 5AZA provided a clearer picture. 5AZA induced site-specific changes in DNA methylation for each day in culture: most sites became hypomethylated early in treatment and then became hypermethylated after several days of treatment. Given that *P. soloecismus* eventually undergoes hypomethylation during its growth cycle (and lipid accumulation), it is possible that 5AZA simply induced hypomethylation early on these particular sites to drive the same phenotype. This early hypomethylation pattern due to 5AZA treatment occurred on several genes involved in lipid synthesis and the cell cycle, including on the CpG site within diacylglycerol O-acyltransferase 2. As many as 40% of sites became hypermethylated in response to 5AZA after several days of 5AZA treatment; however, this hypermethylation coincided with lack of cell division in *P. soloecismus* cultures. It is unlikely that 5AZA interfered with *de novo* methylation; thus, the reversal in the methylation pattern was likely due to *lack* of efficacy of 5AZA given that mitosis had ceased. A subset of genes involved in fatty acid synthesis and elongation have CpG sites and were hypomethylated by 5AZA treatment on Day 6. These included the very-long-chain enoyl-CoA reductase (TER), which catalyzes the last of the four reactions of the long-chain fatty acids elongation cycle; DGAT1, an enzyme that catalyzes the terminal and only committed step in triacylglycerol synthesis by using diacylglycerol and fatty acyl CoA as substrates; and phosphoglycolate phosphatase, which regulates the cellular levels of glycerol-3-phosphate (a metabolic intermediate of glucose) and thus lipid and energy metabolism (Mueller et al., 2017). Thus, while we did not observe global hypomethylation in response to 5AZA treatment, these site specific changes may have been sufficient to alter phenotype.

One of the more interesting findings in this study was the significant hypomethylation of CpG and CHG sites located within genes encoding for chromatin modifying enzymes. These included histone methyltransferase and demethylases, histone acetyltransferases and deacetylases, and the SWI/SNF chromatin remodeling complex. Histone modifications have yet to be measured in *P. soloecismus*; however, the data suggests that in addition to DNA methylation, *P. soloecismus* may use histone modifications. The enzymes responsible for histone modifications are themselves regulated by DNA methylation and responsive to environmental conditions during growth of this species. Indeed, 5AZA treatment altered the methylation ratios of many of these sites within chromatin modifying genes. Several studies demonstrate the importance of histone modifications in regulation of the life cycle and even lipid metabolism in *C. reinhardtii* (Waterborg et al., 1995; van Dijk et al., 2005; Casas-Mollano et al., 2007; Ngan et al., 2015). Our ongoing efforts in analyzing genomic regulation of *P. soloecismus* will explore these mechanisms as well.

In addition to altering methylation ratios on specific CpG sites, 5AZA treatment, remarkably, impacted the phenotype of *P. soloecismus* during the growth cycle. Significant increases in optical density, cell size, cell complexity, and accumulation of lipid biomolecules resulted from 5AZA treatment. 5AZA did not statistically impact cell proliferation, though the variance in this measurement was large. Given the limited number of studies on the effects of 5AZA in microalgae cultures (Xue et al., 2019), it is difficult to put these findings into context. To our knowledge this is the first report of repeated treatments with 5AZA for any algae species. In microalgae cultivation, an increase in optical density, cell size, and cell complexity accompanied by a lack of cellular proliferation, suggests that cellular composition has changed. Using an established flow cytometry assay for assessing lipid content (Steadman Tyler et al., 2019), we measured a significant increase in lipids in the 5AZA treated cultures, beginning on Day 6 in culture. This increase was as much as 66% by Day 8 in culture. Lipid accumulation is a hallmark phenotype that algal researchers seek in selecting a biofuel platform species. Potentially, this finding has far reaching implications, suggesting that manipulation of DNA methylomes (and perhaps other epigenetic modifications) could drive microalgae phenotypes toward any desired feature, including lipid accumulation.

## Conclusion

We sought to determine the role DNA methylation plays in regulating growth and lipid accumulation of *P. soloecismus*, a promising algal biofuel production species. We found genomic sequences for putative DNA methyltransferase enzymes, and initially measured low, but adaptable, 5mC levels. WGBS revealed that approximately 1.15% of the *P. soloecismus* genome contains cytosine methylation in all three contexts, localized to several genomic regions, with approximately 12.1% CpG methylation. The genome becomes hypomethylated across the algal growth cycle, suggesting that nutrient deprivation has an impact on epigenetic regulation of the *P. soloecismus* genome. DNA methylation was further altered by treatment with a DNA methyltransferase inhibitor, 5AZA, across the growth cycle. Hypomethylation of site-specific CpGs in genes involved in fatty acid synthesis and the cell cycle correlated with changes in phenotype, including larger cell size and complexity and accumulation of lipids. Potentially, DNA methylation regulates the cellular response to environmental stressors, such as nitrogen limitation, resulting in carbon sequestration into lipid biomolecules; deeper molecular investigation is needed to assess the validity of this assertion. This is the first report on manipulation of epigenetic mechanisms in algae for the purposes of enhanced biofuel production.

## Data Availability Statement

All datasets presented in this study are included in the article/Supplementary Material can be found on FigShare (see text footnote 3).

## Author Contributions

CSt, SB, CSa, BM, and ST conceived the research and designed the experiments. CSt, YK, and CSa performed the experiments. CSt and SB analyzed the data. CSt performed the statistical analysis and wrote the manuscript. SB, YK, CSa, BM, and ST critically revised and edited the manuscript. CSt, BM, and ST obtained the funding. All authors read and approved the final manuscript.

## Conflict of Interest

The authors declare that the research was conducted in the absence of any commercial or financial relationships that could be construed as a potential conflict of interest.

## Footnotes

1 http://www.bioinformatics.babraham.ac.uk/projects/bismark/

2 https://github.com/dpryan79/MethylDackel/

3 https://figshare.com/authors/Christina_Steadman/8855753

4 https://greenhouse.lanl.gov

5 https://github.com/lanl/DNA_methylation_analysis

## Supplementary Material

The Supplementary Material for this article can be found at: https://www.frontiersin.org/articles/10.3389/fgene.2020.560444/full#supplementary-material and on FigShare at: https://figshare.com/authors/Christina_Steadman/8855753

Supplementary FIGURE 1 Global DNA methylation of *P. soloecismus* determined by ELISA. *P. soloecismus* gDNA percent 5mC was determined using an antibody-based ELISA. *P. soloecismus* gDNA contains 0.82% 5mC content, while *P. soloecismus* gDNA treated for 12 h with CpG methylase has 1.3% 5mC content (*p* < 0.0001); comparison done using Student’s *t*-test. Data are presented as mean ± SEM (standard error of the mean).

Supplementary FIGURE 2 Dose response of *P. soloecismus* after treatment with 5AZA. *P. soloecismus* shaker cultures were treated daily 4–5 h into the light cycle with 0, 5, 10, and 20 μM 5-aza-2′deoxycycdine (5AZA). Prior to treatment each day, optical density (OD_750nm_) was assessed to track growth. 20 μM 5AZA induced the greatest change in optical density of the cultures after 4 days of treatment.

Supplementary FIGURE 3 Top CpG sites with the largest methylation ratio differences between control and 5AZA treated cultures on Day 4. These sites remain hypomethylated through the time course. Average methylation ratios are shown for control and 5AZA treated cultures on Day 4. Methylation ratio differences between control and 5AZA treated cultures on Day 4 are labeled as METHYL DIFF and are significant (*p* < 0.05). Annotations (NSC_ID corresponding to the *P. soloecismus* genomic ID) and the KO (KEGG Orthologies, within E-24) definitions are provided.

Supplementary TABLE 1 All cytosine sites called from whole genome bisulfite sequencing analysis (WGBS).

Supplementary TABLE 2 All significantly hypomethylated CpG sites for all days in culture.

Supplementary TABLE 3 All significantly hypermethylated CpG sites across all days in culture with KO annotations.

Supplementary TABLE 4 Hypomethylated CpG sites belonging to specific KO pathways.

Supplementary TABLE 5 Day 4 in culture with 5AZA treatment; all significantly hypomethylated and hypermethylated CpG sites.

Supplementary TABLE 6 Day 5 in culture with 5AZA treatment; all significantly hypomethylated and hypermethylated CpG sites.

Supplementary TABLE 7 Day 6 in culture with 5AZA treatment; all significantly hypomethylated and hypermethylated CpG sites.

Supplementary TABLE 8 Day 7 in culture with 5AZA treatment; all significantly hypomethylated and hypermethylated CpG sites.

Supplementary TABLE 9 Day 10 in culture with 5AZA treatment; all significantly hypomethylated and hypermethylated CpG sites.
