# Supplementary figures and images for: Inhibition of DNA Methylation in Picochlorum soloecismus Alters Algae Productivity
Source: Front Genet. 2020 Oct 15;11:560444. doi: 10.3389/fgene.2020.560444 (PMC7593850; doi:10.3389/fgene.2020.560444)

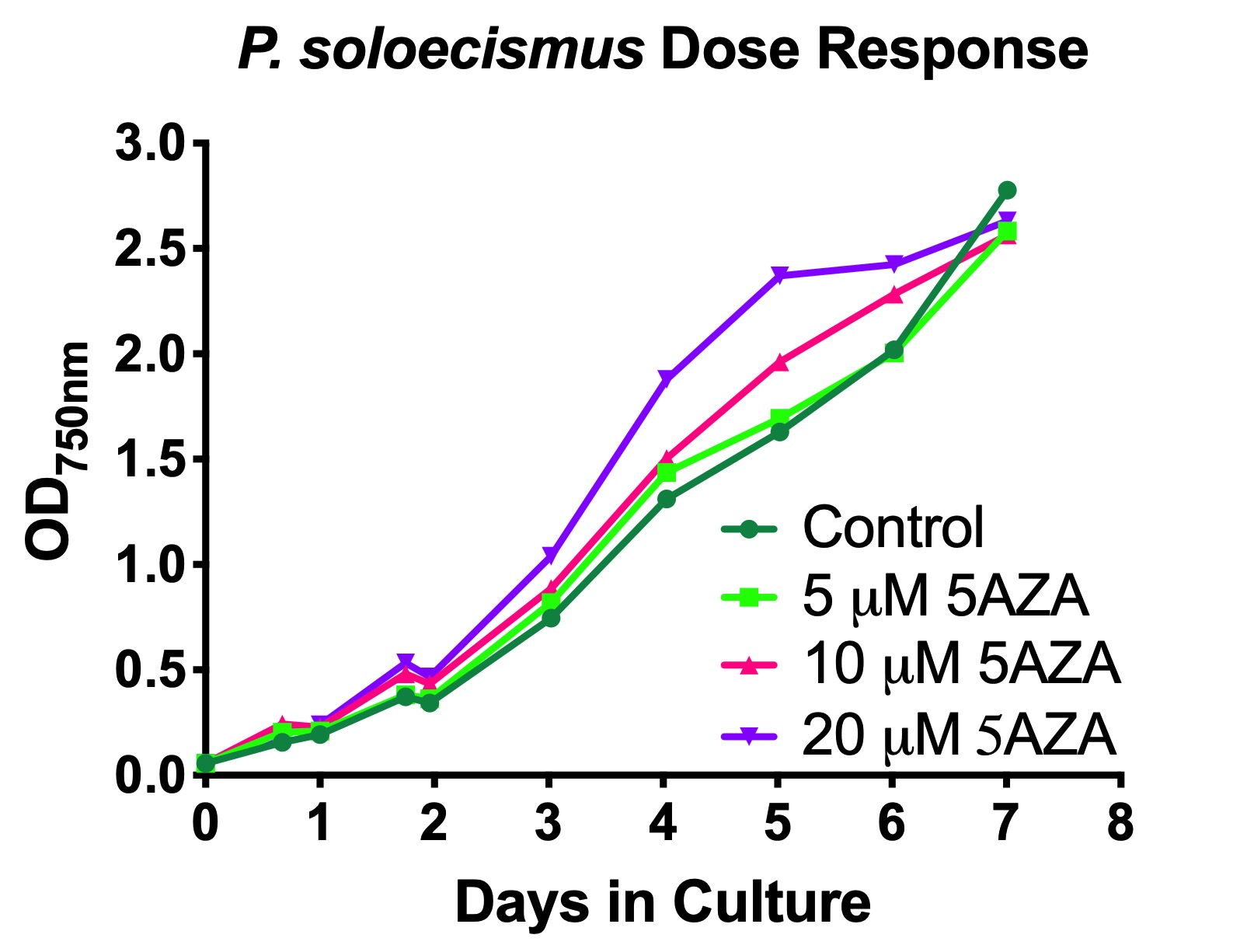

Supplement: Supplementary FIGURE 1 — Global DNA methylation of P. soloecismus determined by ELISA. P. soloecismus gDNA percent 5mC was determined using an antibody-based ELISA. P. soloecismus gDNA contains 0.82% 5mC content, while P. soloecismus gDNA treated for 12 h with CpG methylase has 1.3% 5mC content (p < 0.0001); comparison done using Student’s t-test. Data are presented as mean ± SEM (standard error of the mean). [file Image_1.jpeg]

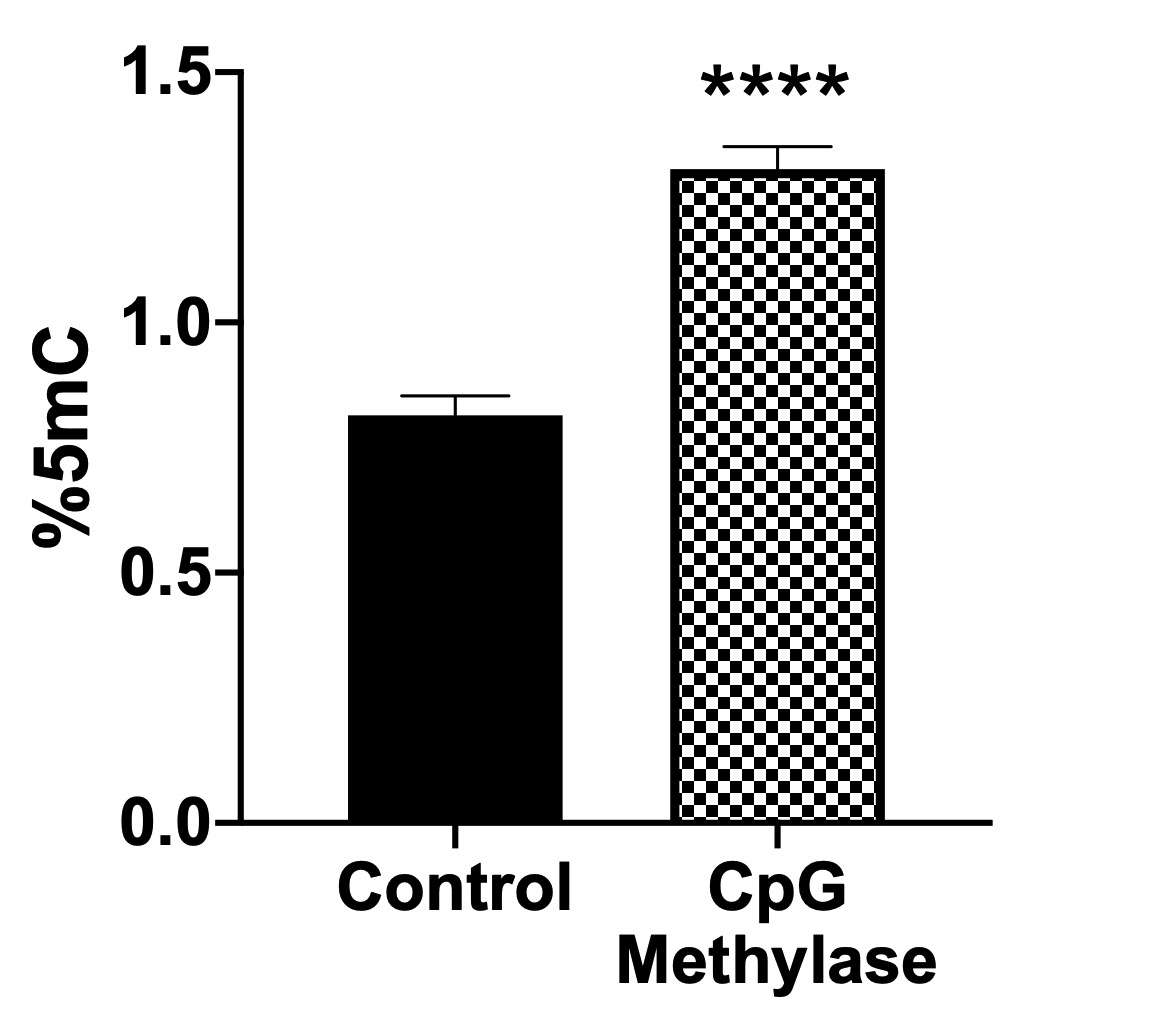

Supplement: Supplementary FIGURE 2 — Dose response of P. soloecismus after treatment with 5AZA. P. soloecismus shaker cultures were treated daily 4–5 h into the light cycle with 0, 5, 10, and 20 μM 5-aza-2′deoxycycdine (5AZA). Prior to treatment each day, optical density (OD750nm) was assessed to track growth. 20 μM 5AZA induced the greatest change in optical density of the cultures after 4 days of treatment. [file Image_2.jpeg]

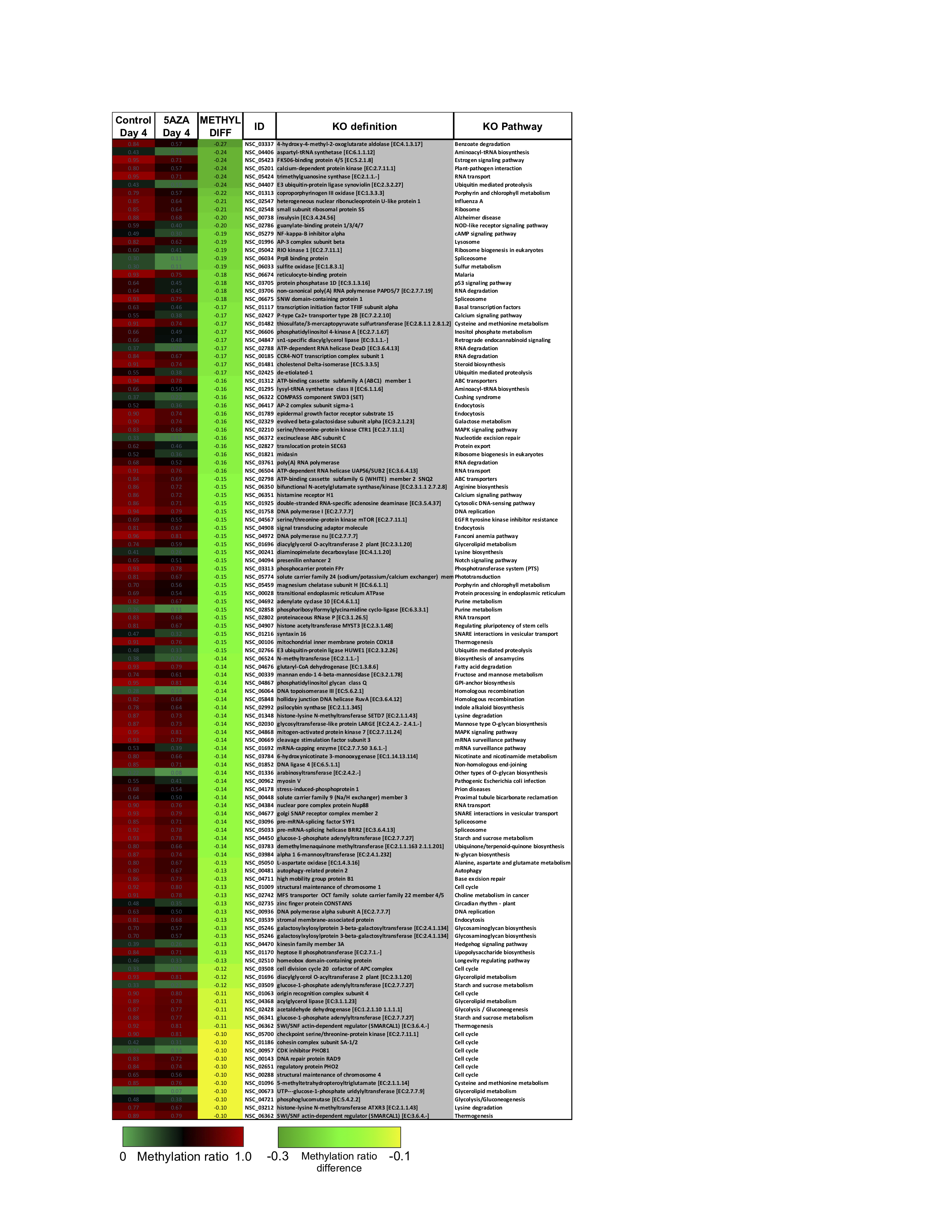

Supplement: Supplementary FIGURE 3 — Top CpG sites with the largest methylation ratio differences between control and 5AZA treated cultures on Day 4. These sites remain hypomethylated through the time course. Average methylation ratios are shown for control and 5AZA treated cultures on Day 4. Methylation ratio differences between control and 5AZA treated cultures on Day 4 are labeled as METHYL DIFF and are significant (p < 0.05). Annotations (NSC_ID corresponding to the P. soloecismus genomic ID) and the KO (KEGG Orthologies, within E-24) definitions are provided. [file Image_3.jpeg]
